# How should long-term free-living physical activity be targeted after stroke? A systematic review and narrative synthesis

**DOI:** 10.1186/s12966-018-0730-0

**Published:** 2018-10-17

**Authors:** Sarah A. Moore, Nina Hrisos, Darren Flynn, Linda Errington, Christopher Price, Leah Avery

**Affiliations:** 1 NIHR Newcastle Biomedical Research Centre based at Newcastle upon Tyne Hospitals NHS Trust and Newcastle University, Newcastle upon Tyne, UK; 2 Institute of Health and Society, Newcastle University, Newcastlee Upon Tyne, NE2 4AX UK; 3 Faculty of Medical Sciences, Newcastle University, Newcastle upon Tyne, NE2 4HH UK; 4 School of Health & Social Care, Centuria Building, Teesside University, Middlesbrough, TS1 3BX UK

**Keywords:** Stroke, Physical activity, Sedentary behaviour, Systematic review, Behaviour change

## Abstract

**Background:**

Increasing physical activity (PA) levels (regular movement such as walking and activities of daily living) and reducing time spent sedentary improves cardiovascular health and reduces morbidity and mortality. Fewer than 30% of independently mobile stroke survivors undertake recommended levels of PA. Sedentary behaviour is also high in this population. We aimed to systematically review the study characteristics and the promise of interventions targeting free-living PA and/or sedentary behaviour in adult stroke survivors.

**Methods:**

Seven electronic databases were searched to identify randomised controlled trials (≥3-months follow-up) targeting PA and/or sedentary behaviour in adults with first or recurrent stroke or transient ischaemic attack. The quality assessment framework for RCTs was used to assess risk of bias within and across studies. Interventions were rated as “very”, “quite” or “non-promising” based on within- or between-group outcome differences. Intervention descriptions were captured using the TIDieR (Template for Intervention Description and Replication) Checklist. Behaviour change techniques (BCTs) within interventions were coded using the BCT Taxonomy v1, and compared between studies by calculating a promise ratio.

**Results:**

Nine studies fulfilled the review criteria (*N* = 717 randomised stroke patients) with a high or unclear risk of bias. None of the studies targeted sedentary behaviour. Six studies were very/quite promising (reported increases in PA post-intervention). Studies were heterogeneous in their reporting of participant age, time since stroke, stroke type, and stroke location. Sub-optimal intervention descriptions, treatment fidelity and a lack of standardisation of outcome measures were identified. Face to face and telephone-based self-management programmes were identified as having promise to engage stroke survivors in PA behaviour change. Optimal intensity of contact, interventionist type and time after stroke to deliver interventions was unclear. Nine promising BCTs (ratios ≥2) were identified: information about health consequences; information about social and environmental consequences; goal setting-behaviour; problem-solving; action planning; feedback on behaviour; biofeedback; social support unspecified; and credible source.

**Conclusions:**

Future research would benefit from establishing stroke survivor preferences for mode of delivery, setting and intensity, including measurement of physical activity. Interventions need to justify and utilise a theory/model of behaviour change and explore the optimal combination of promising BCTs within interventions.

**Electronic supplementary material:**

The online version of this article (10.1186/s12966-018-0730-0) contains supplementary material, which is available to authorized users.

## Background

Increasing physical activity levels (regular movement such as walking and activities of daily living) and reducing time spent sedentary improves cardiovascular health and reduces morbidity and mortality [1, 2]. Fewer than 30% of independently mobile stroke survivors undertake recommended levels of physical activity [3, 4]. Time spent sedentary is high after stroke, with individuals spending up to 22 h a day sitting or lying down [5]. Increasing physical activity and reducing sedentary behaviour after stroke can improve walking ability and balance [6], control risk factors associated with further cardiovascular disease [7] and attenuate low mood and social isolation frequently observed after stroke [8, 9]. Targeting physical activity and sedentary behaviour after stroke is complex due to stroke-related impairments; lack of professional support; poor information provision; cost and access to resources; and reduced self-efficacy for engaging in physical activity [10, 11].

There is a pressing need to develop and implement interventions that address barriers to long-term engagement in physical activity. Structured exercise programmes targeting physical fitness after stroke have been shown to improve short-term physical function [6], cardiorespiratory fitness [12] and metabolic risk factors [7, 13], however the impact of these interventions on free-living physical activity and sedentary behaviour over time has not been established. Structured supervised exercise sessions often have little or no emphasis on free-living physical activity or sedentary behaviour outside of the clinical setting. Consequently, they do not equip individuals with the knowledge, skills and confidence for maintaining increased physical activity over time.

Individualised supported self-management programmes have shown potential for improving participation in everyday activities and functional ability after stroke [14]. The feasibility of applying these approaches to post-stroke physical activity has been recently tested in a number of small studies with favourable results [15, 16]; however their efficacy has yet to be established. A review of interventions targeting long-term physical activity [17] indicated that ‘tailored counselling’ may lead to improved long-term physical activity outcomes after stroke. The effectiveness of these interventions, however, was not established and the behavioural techniques used during the tailored counselling sessions were not reported or defined using a standardised taxonomy.

The application of psychological theory is recommended for the development of complex behavioural interventions [18] and when fully operationalised can increase their effectiveness [19]. Utilisation and specification of behaviour change techniques (BCTs) within interventions facilitates operationalisation of psychological theory, enabling a clearer understanding of which intervention components are associated with effective changes in target behaviour [20]. This methodology has been used previously to inform the design of free-living physical activity interventions in long-term conditions [21, 22]. Replication of this methodology incorporating a framework such as the Template for Intervention Description and Replication (TIDieR) [23] to systematically report intervention content would enable the development of novel interventions in the context of physical activity and stroke. A systematic development process would facilitate the development of interventions that have the potential for engaging stroke survivors in making choices about the type and intensity of physical activity that are consistent with their individual needs and preferences.

We aimed to systematically review the study characteristics and the promise of interventions targeting free-living physical activity and/or sedentary behaviour in adult stroke survivors, in order to inform the design of a novel theory- and evidence-based intervention.

## Review methods

We adhered to a protocol [24] and the Preferred Reporting Items for Systematic Reviews and Meta-Analyses (PRISMA) guidelines [25]. A PRISMA checklist is provided in Additional file 1.

### Review criteria

Randomised controlled trials of interventions targeting free-living physical activity and/or sedentary behaviour (as either the primary or secondary outcome) of adults aged ≥18 years diagnosed with first or recurrent stroke or transient ischaemic attack were eligible for inclusion. Interventions targeting multiple lifestyle behaviours (e.g. physical activity, diet and smoking cessation) were included if they provided a clear description and outcome relating to the physical activity and/or sedentary behaviour. Studies also had to report on changes in free-living physical activity and/or sedentary behaviour measured in terms of frequency and/or duration and/or intensity, either objectively (e.g. accelerometer) or subjectively (i.e. self-reported measures such as questionnaires) at least 3-months post-intervention. Interventions delivered by healthcare and non-healthcare professionals (including remotely by the internet or telephone) within inpatient, early supported discharge, outpatient and community settings were eligible for inclusion. Comparator groups eligible for inclusion were usual care or comparator interventions without a physical activity/sedentary behaviour component (e.g. social and educational sessions).

Studies were excluded if they were conducted in an inpatient setting or exercise laboratory where participants were not encouraged to engage in free-living physical activity or to reduce their time spent sedentary when discharged; only targeted the upper limb(s); or focused on an assistive gait device (e.g. ankle foot orthosis, walking stick, robotics). Pharmaceutical, transcranial magnetic stimulation and treadmill training interventions, unless the comparator arm was a physical activity/sedentary behaviour-based intervention, were also excluded.

### Search strategy

Seven electronic databases were searched up to February 15th 2017: PsycINFO; MEDLINE; CINAHL; EMBASE; Scopus; the Cochrane Library and Web of Science. The search strategy was designed and conducted by an Information Specialist (LE). An example search strategy applied within MEDLINE can be found in Additional file 2. No restrictions were placed on date of publication or language. Reference lists of included studies were hand searched. Citation searches of included studies were undertaken using ISI Web of Science.

### Study selection

Two reviewers (SM/NH) independently screened titles and abstracts retrieved by the search strategy. Full-text articles were then reviewed independently by the same two reviewers using a study selection form. Any disagreements were resolved via discussion. If agreement was not reached, then a third reviewer was asked to adjudicate (DF/LA).

### Data extraction

A standardised data extraction form was developed and piloted on one study (Additional file 3). Two reviewers (SM/NH) independently extracted data from retained full text studies. Any disagreements were resolved via discussion or adjudicated by a third member of the review team (DF/LA). Inter-rater reliability of data extraction was calculated as percentage agreement between coders. Missing data was sought by contacting the corresponding authors of included studies.

Data were extracted on: setting; study population; comparator arm(s); intervention content (description, theory and theory-linked BCTs); changes in physical activity/sedentary behaviour; and assessment periods.

Intervention descriptions were captured using the TIDieR (Template for Intervention Description and Replication) Checklist [23], including categories “brief name”, “why”, “what (materials)”, “what (procedures)”, “who provided”, “how”, “where”, “when and how much”, “tailoring and modifications”, “how well (actual)” and “how well (planned)”.

Theoretical underpinning of interventions (specific theory and operationalisation), where explicitly stated, was extracted using a revised version of the Theory Coding Scheme [26].

BCTs used within interventions were extracted by two reviewers (SM/NH) trained in the use of the Behaviour Change Technique Taxonomy v1 [20]. BCTs identified within both the intervention and control arms of included studies were discounted from analyses.

### Risk of bias

The methodological quality assessment framework for RCTs [27] was used independently by two reviewers (SM,NH) to assess the risk of bias within and across studies. Risk of bias for each study was graded as “low,” “high” or “unclear” for each category.

### Intervention promise

Due to heterogeneity in mode of delivery, intervention content and outcomes, a meta-analysis was inappropriate and inconsistent with the aims of the review. We present a narrative synthesis of the content and promise of behavioural interventions (based on criteria used in previous reviews that have investigated intervention components in relation to promise [28, 29]), in order to inform the development of a new intervention.

Interventions were grouped into three categories of ‘promise’ relating to their potential (statistically significant within- or between-group) increases in outcomes at one or more follow-up points relative to baseline: very promising (statistically significant between-group improvements in outcomes in favour of the intervention group); quite promising (intervention groups showed statistically significant within-group improvements in outcomes, or improvements greater than those in a comparator group); and non-promising (no statistically significant improvements in outcomes either within or between groups).

### Quality of reporting on intervention content

Each category with TiDieR was coded as adequately reported (score = 1) or inadequately reported/absent (score = 0). A score of 1 was assigned if specific categories were not applicable. Total scores out of a maximum of 12 points are reported as percentages. Intervention content extracted using TIDieR were described in relation to intervention promise.

### Fidelity of intervention delivery

Data were extracted on treatment fidelity measures using checklist consisting of 16 items developed with reference to published guidance [30]. An example checklist is presented in Additional file 3. One point was given for each fidelity item that studies employed. A score of zero was given if a measure was not explicitly described by the study authors. A total fidelity score (out of 16) was calculated along with a percentage of the total score.

### Promise of BCTs

The potential of BCTs within interventions for changing the desired behaviour was assessed with a “promise ratio” for each BCT. This was calculated by summing the very or quite promising interventions featuring a specific BCT and dividing this by the number of non-promising interventions featuring the same BCT. BCTs found in at least twice as many promising (very or quite) as non-promising interventions (promise ratio of ≥2) were classified as promising BCTs [28].

BCTs found in two or more promising interventions, but not in any non-promising interventions (promise ratio of 0) were reported as the number of promising interventions in which a BCT featured.

## Results

**Fig. 1:**
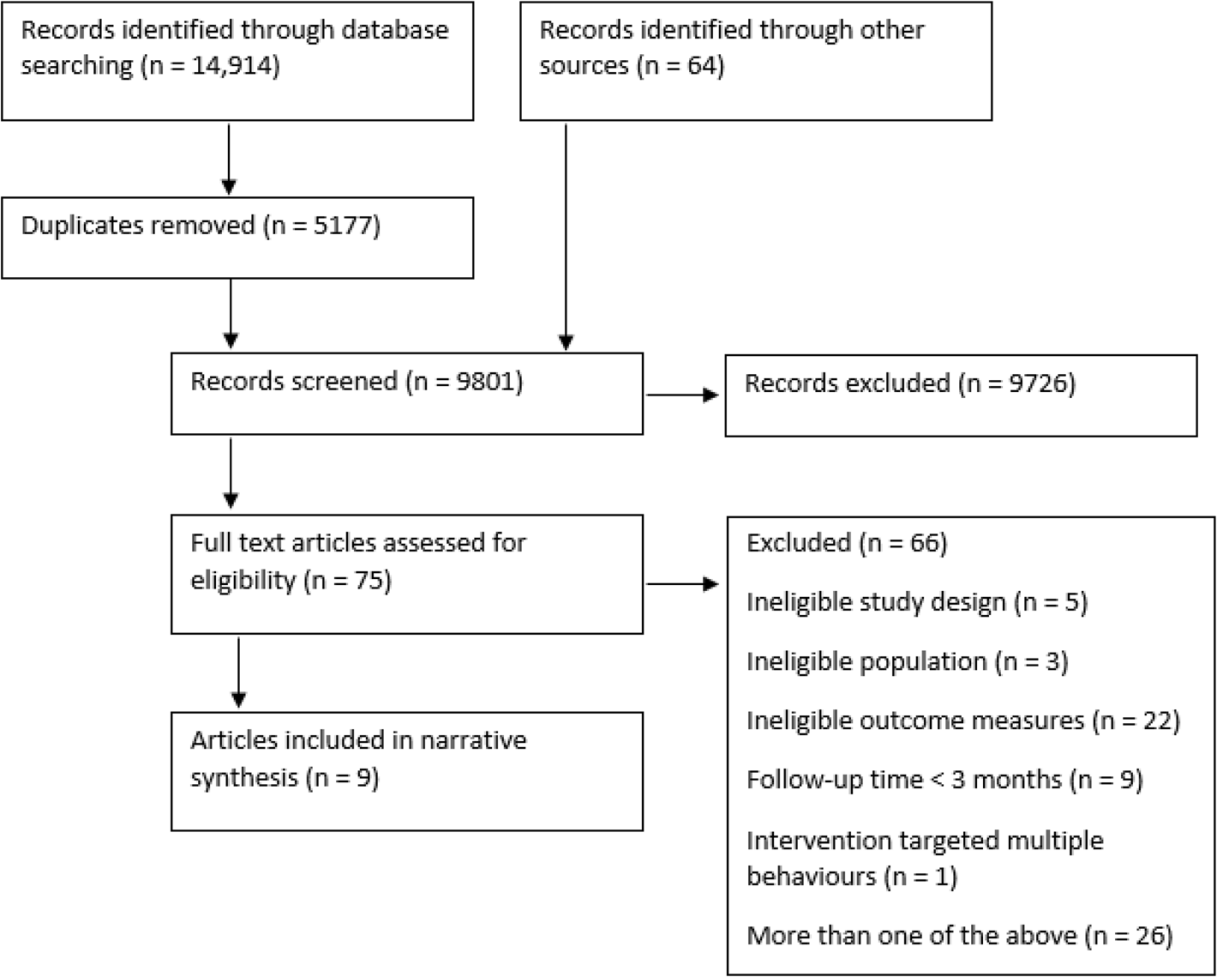
PRISMA flow-chart

A total of 9801 references were returned from the search strategy after removal of duplicates (Fig. 1). 75 articles were identified as potentially relevant, with 9 studies fulfilling all review criteria [31–39]. For a list of excluded studies see Additional file 4. All 9 included studies focused on physical activity interventions. None reported on sedentary behaviour.

### Study characteristics

Study characteristics, methodological quality, outcome measures and changes in physical activity outcomes are presented in Table 1. Across all nine included studies there were 719 randomised adult stroke survivors (ranging from 20 to 190; median = 74) of which 59% were male. The mean age of intervention participants was 64.5 years (65.3 years for controls/comparison groups). Studies were heterogeneous in their reporting of participant age, length of time since stroke, stroke type, and stroke location. One study [35] had three arms (two intervention arms and an attention matched control arm that performed an upper limb training programme). The remaining eight studies [31–34, 36–39] had two arms (intervention versus usual care/attention matched control).

**Table 1:** Summary of study characteristics, quality measures and outcomes

Study	Study characteristics	Quality measures	Intervention (brief description)	Control (brief description)	Primary outcome measure	PA outcome measures	Changes in PA outcome measures
*Very promising*							
*Olney* et al. *2006* [31]	*Sample size:* *N* = 74 IG: 38, CG: 36 *Gender:* M: 45 (62.5%), F: 27 (37.5%) *Mean (SD) age in years:* IG: 63.5 (12) CG: 65.8 (11.6) *Follow-up times:* 10 weeks, 6 months & 1 year	*Fidelity score:* 3/16 (19%) *Methodological quality ratings:* Low risk: 5 Unclear risk: 2 High risk: 3	10-week supervised strengthening and conditioning programme	1-week supervised exercise followed by a 9-week unsupervised home exercise programme	Six-minute walking speed (function)	Human Activity Profile (adjusted activity score)	At 12 months, there was a significant increase in PA in IG compared to CG (*p* < 0.05)
*Quite promising*							
*Damush* et al. *2011* [32]	*Sample size:* *N* = 63 IG: 30, CG: 33 *Gender:* M: 62 (98%); F: 1 (2%) *Mean (SD) age in years:* IG: 67.3 (12.4) CG: 64 (8.4) *Follow-up times:* Baseline, 3 months and 6 months	*Fidelity score:* 6/16 (38%) *Methodological quality ratings:* Low risk: 4 Unclear: 3 High risk: 1	12-week stroke self-management program focusing on increasing self-efficacy	Stroke-related education materials & pamphlets on secondary stroke prevention. 6 bi-weekly telephone calls for 12 wks, during which participants were asked how they were doing that day	Stroke-specific quality of life, assessed using the SSQOL (quality of life)	Frequency of exercise behaviour within the past week measured using validated scale	At 3 and 6 months, PA increased in IG compared to CG (no significant difference)
*Ludwig* et al. *2016* [33]	*Sample size:* *N* = 20 Group specific information unavailable *Gender:* information unavailable *Mean age in years:* 51.5 SD unavailable *Follow-up times:* 4 weeks, 3 months and 6 months after rehabilitation	*Fidelity score:* 9/16 (56%) *Methodological quality ratings:* Low risk: 2 Unclear risk: 4 High risk: 2	A theory-based training session delivered after a course of Nordic walking	Control group participants were invited to a single face-to-face training session based on positive gain and a power point presentation on the health benefits of physical activity	Walking-training frequency and duration of each set per week, using questionnaire (PA behaviour)	Walking-training frequency and duration of each set per week, using questionnaire	Stroke patients in IG showed tendency toward increased PA levels compared to those in CG (no significant difference)
*Morén* et al. *2016* [34]	*Sample size:* *N* = 88 IG: 44, CG: 44 *Gender:* M: 41 (47%); F: 47 (53%) *Mean age (SD) in years:* IG: 69.9 (9.1) CG: 72.3 (8.3) *Follow-up times:* 3 months and 6 months	*Fidelity score:* 7/16 (44%) *Methodological quality ratings:* Low risk: 6 Unclear risk: 2 High risk: 1	Delivery of Physical activity Prescription (PaP)	Usual care	MVPA assessed by Actigraph activity monitor worn on back (PA behaviour)	MVPA and steps per day, assessed by Actigraph activity monitor worn on back	At 6 months, IG showed tendency towards an increase in steps per day compared to CG (no significant difference)
*Severinsen* et al. *2014* [35]	*Sample size:* *N* = 43 IG1: 13, IG2: 14, CG: 16 *Gender:* M: 31 (72%); F: 12 (28%) *Median (IQR) age in years:* IG1: 69 (50–80) IG2: 68 (57–78) CG: 66 (52–80) *Follow-up times:* 1 year	*Fidelity score:* 5/16 (31%) *Methodological quality ratings:* Low risk: 3 Unclear risk: 2 High risk: 3	Aerobic training (IG 1) or progressive resistance training (IG 2)	Low-intensity sham training	6-min walking distance and fast 10-min walking speed (function)	Physical Activity Scale scores expressed as metabolic equivalents	All groups showed significant increases in PA at study end (*P* < 0.05). No significant between-group difference
*Wan* et al. *2016* [36]	*Sample size:* *N* = 91 IG: 46, CG: 45 *Gender:* M: 57 (71%); F: 23 (29%) *Mean age (SD) in years:* IG: 59.07 (12.36) CG: 60.24 (12.57) *Follow-up times:* 3 months and 6 months	*Fidelity score:* 6/16 (38%) *Methodological quality ratings:* Low risk: 6 Unclear risk: 1 High risk: 1	Goal-setting telephone follow-up program	Usual care and educational stroke brochures (IG & CG)	Health behaviour assessed using the Health Promoting Lifestyle Profile II^3^ (lifestyle behaviour)	8 item physical activity subscale of the Health Promoting Lifestyle Profile II	PA increased significantly in all groups at 3 and 6 months (*P* < 0.01) but no significant between-group difference
*Non-promising*							
*Katz-Leurer* et al. *2003* [37]	*Sample size:* *N* = 92 IG: 46, CG: 46 *Gender:* M: 50 (54%); F: 42 (46%) *Mean age (SD) in years:* 63 (11) Group age characteristics unavailable *Follow-up times:* 6 months	*Fidelity score:* 5/16 (31%) *Methodological quality ratings:* Low risk: 3 Unclear risk: 4 High risk: 1	Early aerobic training	Usual care	Physical fitness measured by a graded stress test performed on a cycle ergometer (physical fitness)	Independence in daily and social activities, using the Frenchay Activities Index	No improvements in PA observed in IG or CG
*Mudge* et al. *2009* [38]	*Sample size:* *N* = 58 IG: 31, CG: 27 *Gender:* M: 32 (55%); F: 26 (45%) *Age:* data unavailable*Follow-up times:* 3 months	*Fidelity score:* 12/16 (75%) *Methodological quality ratings:* Low risk: 6 Unclear risk: 1 High risk: 1	Circuit exercise based rehabilitation	Attention-matched social and educational sessions	Mean number of steps per day measured with StepWatch activity monitor (PA behaviour)	Mean number of steps per day as measured by the StepWatch activity monitor, PADS score	No improvements in PA observed in IG or CG
*Sit* et al. *2007* [39]	*Sample size:* *N* = 190 IG: 107, CG: 83 *Gender:* M: 105 (55%); F: 85 (45%) *Mean age (SD) in years:* IG: 63.5 (12) CG: 65.8 (11.6) *Follow-up times:* Baseline, 1 week, 3 months	*Fidelity score:* 9/16 (56%) *Methodological quality ratings:* Low risk: 3 Unclear risk: 2 High risk: 3	Educational secondary stroke prevention programme	Usual care and provision of information materials on stroke and stroke prevention	Stroke knowledge; Self health monitoring practice; health behaviours (lifestyle behaviour)	Modified Exercise Scale	No improvements in PA observed in IG or CG

CG, control group; F, female; IG, intervention group; M, male; N, number; PA, physical activity; SD, standard deviation

### Outcome measures

Two studies [34, 38] used objective measures of physical activity (accelerometers). The remaining seven studies [31–33, 35–37, 39] used subjective measures of physical activity. These included self-management exercise behaviour frequency, the Frenchay Activities Index [40], walking training frequency and duration measured through questionnaire, the Human Activity Profile [41], the Physical Activity Scale [42], the modified Exercise Scale [43], and the Health Promoting Lifestyle Profile II (HPLP II) physical activity (8 items) subscale [44]. Physical activity behaviour was the primary outcome in three studies and the secondary outcome in six studies. Other outcomes included walking speed [31, 35], physical fitness [37] and quality of life [32].

### Follow-up assessment periods

Six studies included short- (3-months [32–34, 36, 38, 39]) and medium-term follow-up periods (6-months [31–34, 36, 37]), with only two studies conducting long-term (12 months [31, 35]) follow-up assessments.

### Risk of bias

**Fig. 2:**
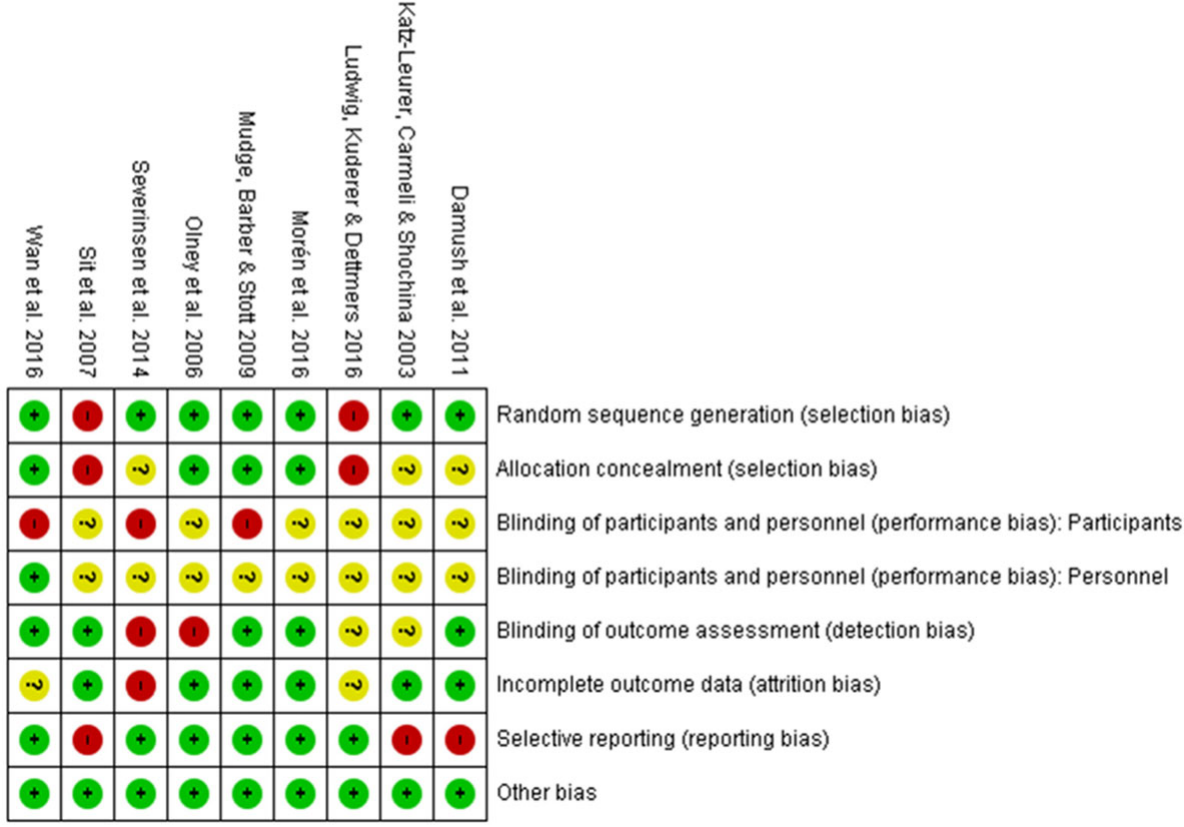
Risk of Bias in included studies

The risk of bias assessment is presented in Fig. 2. Inter-rater coding reliability for risk of bias was 92%, indicating good agreement. All nine studies were rated as having a high or unclear risk of bias for the category “blinding of participants and personnel (performance bias): participants”, which due to inherent difficulties with concealing group allocation is a common feature of behavioural intervention studies.

### Intervention promise

One intervention was rated as very promising [31], five as quite promising [32–36] and three as non-promising [37–39]. Inter-rater reliability for assessment of promise was 89% indicating excellent agreement between coders.

### Quality of intervention reporting

Total scores (out of 12) on the TIDieR checklist for each study are presented in Table 2. Table 3 provides a summary of the frequencies for each TIDieR item according to intervention promise ratings.

**Table 2:** Intervention details described by TIDieR components

Study	Item 1 and 2 TIDieR: Brief name and why (including theory)	Item 3–9 TIDieR^a^: What (materials and procedures), who provided, how, where, when & how much, tailoring	BCTs
Very promising			
*Olney* et al. *2006* [31] *TIDieR score:* 8/12 (67%)	*Brief name:* Face to face structured exercise programme *Why:* It is known that supervised exercise programs improve PA in the short-term but long-term effectiveness has not been established *Theory:* None described	*Materials:* Heart rate monitor, Borg Scale *Procedures:* Structured group exercise programme incorporating warm up, aerobic exercises, strength training, cool down *Who:* Not described *How:* Face to face *Where:* Canada (North America), outpatient rehab centre *When & How much:* 10 weeks, 1.5 h sessions, 3 days/week. Mean time since stroke: > 12 months *Tailoring:* Tailored to each subject’s needs and adjusted weekly as indicated	Self-monitoring of outcome of behaviour, biofeedback, social support (unspecified), instruction on how to perform the behaviour, demonstration of the behaviour, behavioural practice/rehearsal, graded tasks, adding objects to the environment (n = 8)
*Quite promising*			
*Damush* et al. *2011* [32] *TIDieR score:* 12/12 (100%)	*Brief name:* Telephone PA supported self-management *Why:* Most stroke or TIA survivors do not adequately control their stroke risk factors *Theory:* Social Cognitive Theory	*Materials:* None described *Procedures:* Discussions focussing on increasing self-efficacy were conducted *Who:* Nurse, assistant physician, and Master’s level social scientist *How:* By telephone *Where:* USA (North America), veteran outpatient clinics *When & how much:* 12 weeks, 6 bi-weekly sessions. Time since stroke: Participants were recruited < 1 month post stroke and started the intervention on discharge. Exact time post stroke was not described. *Tailoring:* Personalised to levels of self-efficacy	Goal setting (behaviour), problem solving, action planning, review behaviour goal, feedback on behaviour, social support (unspecified), information about health consequences, information about social and environmental consequences, information about emotional consequences, graded tasks, credible source (*n* = 11)
*Ludwig* et al. *2016* [33] *TIDieR score:* 9/12 (75%)	*Brief name:* Face to face PA supported self-management *Why:* Accomplishment planning aids long-term orthopaedic rehabilitation but its applicability to neurological patients is unknown *Theory:* Health Action Process Approach	*Materials:* Written standardised manual *Procedures:* Participants completed a group training programme based on five volitional and motivational strategies: positive gain; planning of training dates; if then plans; anticipation and overcoming obstacles. These were applied to promote the uptake of walking in everyday life. *Who:* Not described *How:* Face to face in groups of 2–5 *Where:* Germany (Europe) *When & how much:* 1 session, 80–90 min. Mean time since stroke: > 12 months *Tailoring:* Action plan tailored to participants	Goal setting (behaviour), problem solving, action planning, self-monitoring of behaviour, social support (unspecified) (*n* = 5)
*Morén* et al. *2016* [34] *TIDieR score:* 9/12 (75%)	*Brief name:* Face to face PA supported self-management *Why:* Physical activity Prescription *(*PaP) has been found to benefit health conditions including metabolic syndrome, which is a risk factor for TIA *Theory:* None described	*Materials:* Oral and written information on stroke and physical inactivity risk factors, accelerometer *Procedures:* PaP was delivered to participants in the intervention group one week after discharge *Who provided:* Physical therapist *How:* Face to face and self-management *Where:* Sweden (Europe) *When & how much:* 1 session, 2 weeks after discharge. Time since stroke: not described *Tailoring:* PaP was based on evidence including: reason for PaP, assessment of current PA level, participant’s own goal, and 1–2 prescribed activities	Goal setting (behaviour), action planning, feedback on behaviour, instruction on how to perform the behaviour, behavioural practice/rehearsal, credible source (*n* = 6)
*Severinsen* et al. *2014* [35] *TIDieR score:* 10/12 (83%)	*Brief name:* Face to face structured exercise programme *Why:* It is unclear whether aerobic and resistance training directly impact ambulation and if changes are maintained in the long-term *Theory:* None described	*Materials:* Cycle ergometer, resistance training machine, digital timing devices, isometric dynamometer, online respiratory gas exchange analyser, heart rate monitor *Procedures:* Participants performed supervised group exercises at training facilities *Who provided:* Physiotherapist *How:* Face to face *Where:* Denmark (Europe), stroke research centre *When & how much:* 12 weeks, 3 times/week, 5 min warm up, 1 h training. Time since stroke: 6–36 months *Tailoring:* Tailored to heart rate and one-repetition maximum	Biofeedback, instruction on how to perform the behaviour, demonstration of the behaviour, behavioural practice/rehearsal (*n* = 4)
*Wan* et al. *2016* [36] *TIDieR score:* 9.5/12 (79%)	*Brief name:* Telephone PA supported self-management *Why:* Many stroke patients do not follow health behaviour guidelines, especially in the long-term. Goal setting and telephone follow-up are effective in other areas but have not been investigated in relation to stroke *Theory:* None described	*Materials:* Educational stroke brochures (IG & CG) *Procedures:* Goal-setting follow-up program delivered by telephone *Who provided:* Nurse *How:* By telephone *Where:* China (Asia), community based *When & how much:* 3 months, 3 telephone calls at 1 week, 1 month and 3 months after discharge, each lasting 15–20 min. Time since stroke: not described *Tailoring:* Patients were involved in the goal setting and action planning process	Goal setting (behaviour), action planning, social support (unspecified), instruction on how to perform the behaviour, information about health consequences, information about social and environmental consequences, credible source (*n* = 7)
Non-promising			
*Katz-Leurer* et al. *2003* [37] *TIDieR score:* 9/12 (75%)	*Brief name:* Face to face structured exercise programme *Why*: To determine the influence of an early exercise programme on functional capacity and long-term activity participation *Theory:* None described	*Materials:* Leg cycle ergometer, heart rate monitor *Procedures:* In addition to usual care, patients trained on a leg cycle ergometer *Who provided:* Physiotherapist *How:* Face to face *Where:* Israel (Asia), inpatient rehab department *When & how much:* 8 weeks; Weeks 1 & 2: 5 times/week, 10 mins/day increasing to 20; Weeks 3–8: 3 times/week, 30 mins/day, 60%/heart rate reserve. Time since stroke: < 1 month *Tailoring:* Tailored to each individual based on initial bike stress test	Action Planning, monitoring of others without feedback, instruction on how to perform the behaviour, demonstration of the behaviour, behavioural practice/rehearsal, graded tasks (n = 6)
*Mudge* et al. *2009* [38] *TIDieR score:* 10/12 (83%)	*Brief description:* Face to face structured exercise programme *Why:* To determine whether gains in function resulting from an exercise based programme translate to home or community environment PA *Theory:* None described	*Materials:* None described *Procedures:* Participants took part in group exercise sessions*Who provided:* not adequately described *How:* Face to face *Where:* New Zealand (Australasia), outpatient clinics *When & how much:* 4 weeks, 3 times/week, 50–60 min sessions with 30 mins of exercise. Time since stroke: > 6 months *Tailoring:* Sessions graded to each participant’s ability and progressed as tolerated	Social support (unspecified), instruction on how to perform the behaviour, demonstration of the behaviour, behavioural practice/rehearsal, graded tasks (*n* = 5)
*Sit* et al.*...... 2007* [39] *TIDieR score:* 9.5/12 (79%)	*Brief description:* Face to face PA supported self-management *Why:* Not described *Theory:* None described	*Materials:* Personal log sheets, pedometer *Procedures:* Educational group sessions were held using teaching, games, experience sharing and experimental learning methods *Who provided:* Nurse *How:* Face to face and self-management *Where:* China (Asia), outpatient community *When & how much:* 8 weeks, 1 session/week, 2 h each, in groups of 10–12. Time since stroke not described *Tailoring:* The programme focused on individual goal setting and action plans	Problem solving, self-monitoring of behaviour, social support (unspecified), instruction on how to perform the behaviour, demonstration of the behaviour, behavioural practice/rehearsal, adding objects to the environment (n = 7)

*BCT*, behaviour change technique; *CG*, control group; *IG*, intervention group; min(s), minutes; *N*, number; *PA*, physical activity; *TIA*, transient ischaemic attack; *PaP,* physical activity prescription;

^a^Item 10 is not displayed in this table as no studies reported any intervention modifications. Items 11 and 12, which measure intervention fidelity, are not displayed, as fidelity is assessed using the criteria defined by (Bellg et al., 2004 [30])

**Table 3:** TIDieR item descriptions in relation to intervention promise

TIDieR item^a^	Description	Very Promising	Quite Promising	Non-promising
*Why (rationale/theory)*	Adequately described		2	
	Not adequately described	1	3	3
*What (materials)*	Borg scale & heart rate monitor	1		
	Personal log sheets & pedometer			1
	Written standardised manual		1	
	Written information & accelerometer		1	
	Educational brochures		1	
	Gym based equipment & heart rate monitor		1	1
	Not adequately described		1	1
*What (procedures)*	Structured exercise sessions	1	1	2
	Group discussions focused on self-efficacy		1	
	Motivational and volitional strategies		1	
	Physical activity Prescription		1	
	Goal-setting telephone follow-up program		1	
	Educational sessions			1
*Who provided*	Nurse, assistant physician & social scientist		1	
	Physiotherapist		2	1
	Nurse		1	1
	Not adequately described	1	1	1
*How (mode of delivery)*	Face to face exercise sessions	1	1	2
	Face to face supported self-management		2	1
	Telephone supported self-management		2	
*Where*	Canada	1		
	USA		1	
	Germany		1	
	Sweden		1	
	Denmark		1	
	China		1	1
	Israel			1
	New Zealand		1	
	Outpatient rehabilitation centre/clinic	1	1	1
	Inpatient rehabilitation centre			1
	Stroke research centre		1	
	Community based		1	1
	Not adequately described		2	
*When & How Much*	Delivered in single session		2	
	Delivered over 4 weeks			1
	Delivered over 8 weeks			2
	Delivered over 10 weeks	1		
	Delivered over 12 weeks		3	
	Delivered in acute stages			1
	Delivered in chronic stages	1	2	1
	Stroke stage not adequately described		3	1
	1 contact over intervention delivery period		2	
	8 contacts over intervention delivery period			1
	12 contacts over intervention delivery period			1
	24 contacts over intervention delivery period		2	
	28 contacts over intervention delivery period			1
	30 contacts over intervention delivery period	1		
	36 contacts over intervention delivery period		1	
*Tailoring*	Tailored to participants	1	5	3
	Not tailored to participants			
*Modifications*	Modifications reported			
	No modifications reported	1	5	3

^a^Items 11 & 12 on intervention fidelity are not displayed (assessed using Bellg et al. 2004 [30]

The median score for all nine interventions, and those rated as quite promising and non-promising was 9.5/12 (IQR = 1). The intervention rated as very promising [31] scored 8/12 on TIDieR categories Of the five quite promising interventions, one [32] scored 12 on TIDieR; one scored 10 [35]; one scored 9.5 [36]; and two scored 9 [33, 34]. The non-promising interventions scored 10 [38], 9.5 [39] and 9 [37] on TIDieR categories.

### Summary of intervention components using the TIDieR framework

#### TIDieR item 1: Brief name

Table 2 provides the brief names of all the included interventions.

#### TIDieR item 2: Why

Two of the nine studies [32, 33], both rated as quite promising, made an explicit reference to a behaviour change theory. The first [33] was developed in accordance with the Health Action Process Approach [45] and explicitly targeted motivation, volition, accomplishment planning and coping planning. The second intervention [32] was developed in accordance with Social Cognitive Theory [46], but targeted only two constructs of the theory: self-efficacy and self-regulation.

#### TIDieR item 3: What (materials)

The very promising intervention [31] used a heart rate monitor and a Borg Scale [47]. One quite promising intervention did not describe any intervention materials used [32]. Three quite promising interventions used paper-based tools: a written standardised manual [33]; oral and written information on stroke and physical inactivity risk factors [34]; and educational stroke brochures in the intervention and control groups [36]. The remaining quite promising intervention [35] used gym-based equipment and physical activity monitoring systems including a cycle ergometer, resistance training machine, digital timing devices, isometric dynamometer, online respiratory gas exchange analyser and a heart rate monitor.

One non-promising intervention [38] did not describe any materials. The remaining two used a leg cycle ergometer and a heart rate monitor [37], and personal log sheets and a pedometer [39].

#### TIDieR item 4: What (procedures)

The very promising intervention [31] used structured exercise sessions beginning with warm up, followed by aerobic exercises, strength training and a cool down period. One quite promising intervention [35] also used supervised group exercise sessions at training facilities. The other 4 quite promising interventions consisted of discussions focusing on increasing self-efficacy [32]; use of motivational and volitional strategies to promote the uptake of walking and maintenance in the long-term [33]; providing participants with a physical activity prescription one week following discharge from hospital [34]; and delivery of a goal-setting follow-up programme over the telephone [36].

The non-promising interventions [37–39] involved leg cycle ergometer training; group exercise sessions; and educational sessions using teaching, games, experience sharing and experimental learning methods.

#### TIDieR item 5: Who provided

The interventionist in the very promising intervention [31] one of the non-promising interventions [38] was not reported. In the quite promising interventions the interventionists were nurses [32, 36]; an assistant physician [32]; a master’s level social scientist [32]; physical therapist [34] and a physiotherapist [35]. In two non-promising interventions the interventionists were a physiotherapist [37] and a nurse [39].

#### TIDieR item 6: How

The very promising intervention used face-to-face structured exercise sessions. Four quite promising interventions used supported self-management delivered face-to-face [33, 34] or telephone [32, 36]. The final quite promising intervention used face-to-face structured exercise sessions [35]. The non-promising interventions [37–39] used face-to-face structured exercise sessions/supported self-management.

#### TIDieR item 7: Where

Studies originated from Canada [31], USA [32], Germany [33], Sweden [34], Denmark [35], China [36, 39], Israel [37] and New Zealand [38].

The very and quite promising interventions were conducted within outpatient rehabilitation centres [31, 32], a stroke research centre [35] or the community [36]. The location where the intervention conducted was not adequately described in two studies [33, 34]. The non-promising interventions were conducted in inpatient [37] or outpatient rehabilitation centre [38], and the community [39].

#### TIDieR item 8: When and how much

The very promising intervention [31] was delivered over 10 weeks. The quite promising interventions were delivered over 12 weeks [32, 35, 36] or a single session [33, 34]. The very promising [31] and two quite promising interventions [33, 35] were delivered during the chronic stages of stroke recovery (> 6 months post-stroke). Three quite promising interventions [32, 34, 36] did not adequately describe the length of time since stroke.

The non-promising interventions were delivered over 4 weeks [38] and 8 weeks [37, 39]. Non-promising interventions were conducted in the acute (< 1 month post-stroke [37]) and chronic stages of stroke recovery (> 6 months post-stroke [38]), and one did not describe the length of time since stroke [39].

Intensity as a function of number of contacts with participants over the intervention delivery periods for very and quite promising interventions were 1 [33, 34], 24 [32, 36], 30 [31] and 36 [35] contacts. For the non-promising interventions the number of contacts were 8 [39], 12 [38] and 28 [37].

#### TIDieR item 9: Tailoring

The very promising intervention [31] was tailored to participants’ ability and adjusted where necessary. The quite promising interventions were tailored to participants’ self-efficacy [32]; reason for physical activity prescription, assessment of current PA level [34]; goals and action plans [33, 34, 36]; and heart rate and one-repetition maximum [35]. The non-promising interventions [37–39] were also tailored based on initial bike stress test results; participant ability; and individuals’ goals and action plans.

#### TIDieR item 10: Modifications

None of the interventions reported any modifications.

#### *TIDieR items 11 and 12 – How well planned and how well actual*treatment fidelity scores are presented in Table 4. Inter-rater reliability for treatment fidelity was 95%

**Table 4:** Treatment fidelity scores of included studies

		Very promising	Quite promising					Non-promising			
		Olney et al. [31]	Damush et al. [32]	Ludwig et al [33]	Morén et al. [34]	Severinsen et al. [35]	Wan et al. [36]	Katz-Laurer et al [37]	Mudge et al [38]	Sit et al. [39]	Total (%)
1) Treatment fidelity strategies for design of study	Ensure same treatment dose within conditions	Y	Y	Y	Y	Y	Y	Y	Y	Y	9 (100%)
	Ensure equivalent dose across conditions	Y	Y	Y	Y	Y	Y	Y	Y	N	8 (89%)
	Plan for implementation setbacks	N	N	N	N	N	N	N	N	N	0 (0%)
2) Treatment fidelity strategies for monitoring and improving provider training	Standardise training	N	Y	N	Y	N	Y	N	Y	N	4 (44%)
	Ensure provider skill acquisition	N	N	N	N	N	Y	N	Y	N	2 (22%)
	Minimise “drift” in provider skills	N	N	N	N	N	N	N	Y	N	1 (11%)
	Accommodate provider differences	N	N	Y	N	N	N	N	Y	N	2 (22%)
3) Treatment fidelity strategies for monitoring and improving delivery of treatment	Control for provider differences	N	N	Y	N	N	Y	N	Y	N	3 (33%)
	Reduce differences within treatment	N	Y	Y	N	N	Y	N	Y	Y	5 (56%)
	Ensure adherence to treatment protocol	N	Y	Y	N	N	N	Y	Y	Y	5 (56%)
	Minimise contamination between conditions	N	Y	Y	Y	Y	N	Y	Y	Y	7 (78%)
4) Treatment fidelity strategies for monitoring and improving receipt of treatment	Ensure participant comprehension	N	N	Y	Y	N	N	N	N	Y	3 (33%)
	Ensure participant ability to use cognitive skills	N	N	N	Y	N	N	N	N	Y	2 (22%)
	Ensure participant ability to perform behavioural skills	Y	N	Y	Y	Y	N	Y	Y	Y	7 (78%)
5) Treatment fidelity strategies for monitoring and improving enactment of treatment skills	Ensure participant use of cognitive Skills	N	N	N	N	N	N	N	N	Y	1 (11%)
	Ensure participant use of behavioural skills	N	N	N	N	Y	N	N	Y	Y	3 (33%)
*Total (out of 16)*		*3*	*6*	*9*	*7*	*5*	*6*	*5*	*12*	*9*	
*Percentage*		*19%*	*38%*	*56%*	*44%*	*31%*	*38%*	*31%*	*75%*	*56%*	

The highest fidelity score assigned to a quite promising intervention was 9/16 [33]; although the highest score on fidelity (12/16) across all nine interventions was for a non-promising intervention [38]. Indeed, the median treatment fidelity score for non-promising interventions [37–39] was 9 out of 16 (IQR = 3.5, range 5–9) compared to 6 out of 16 (IQR = 1, range 6–9) for the quite promising interventions [32–36].

The intervention rated as very promising [31] received a fidelity score of only 3 out 16. It adequately reported ensuring the same treatment dose within across conditions, and participants’ ability to perform behavioural skills. No other treatment fidelity categories were adequately described.

All five quite promising interventions [32–35] reported use of at least one fidelity strategy related to study design (ensuring the same treatment dose within and across conditions). Planning for implementation setbacks was not adequately addressed by any of nine interventions.

Strategies for monitoring and improving interventionist training was addressed by four quite promising interventions [32–34, 36]: providing standardised training to interventionists [32, 34, 36]; ensuring interventionist skill acquisition [36] and accommodating interventionist differences [33]. Minimisation of skill drift in interventionists was not adequately addressed by any very or quite promising interventions, but was a used within one non-promising intervention [38].

All five promising interventions adequately described at least one fidelity strategy for monitoring and improving delivery of treatment: minimising contamination between conditions [32–35]; reducing differences within treatment [32, 33, 36]; controlling for provider differences [33, 36]; and adherence to study protocols [32, 33].

At least one fidelity strategy for monitoring and improving receipt of treatment was used by three promising interventions: ensuring participant comprehension [33, 34]; ensuring participant ability to use cognitive skills [34]; and ensuring participant ability to perform behavioural skills [33–35].

Only one promising intervention [35] adequately ensured participant use of behavioural skills as a fidelity strategy to monitor and improve enactment of treatment skills. Two of three non-promising interventions also employed this strategy [38, 39] as well as ensuring participant use of cognitive skills [39].

### Behaviour change techniques (BCTs)

Inter-rater reliability for coding of the BCTs was 98%. Nineteen different BCTs were identified across the nine studies (Table 5). The median number of BCTs used across all nine interventions was 6 (IQR = 2). The very promising intervention included 8 BCTs (Table 2); the quite promising interventions between 4 and 11 (median = 6, IQR = 2); and the non-promising interventions between 5 and 7 (median = 6, IQR = 1).

**Table 5:** Ratio of BCTs to promise

BCT	Times used	Presence in very/quite interventions containing	Presence in non-promising interventions	Ratio
1. Action planning	5	4	1	4.00
2. Goal setting (behaviour)	4	4	0	4.00
3. Credible source	3	3	0	3.00
4. Social support (unspecified)	6	4	2	2.00
5. Problem solving	3	2	1	2.00
6. Biofeedback	2	2	0	2.00
7. Feedback on behaviour	2	2	0	2.00
8. Information about health consequences	2	2	0	2.00
9. Information about social & environmental consequences	2	2	0	2.00
10. Instruction on how to perform the behaviour	7	4	3	1.33
11. Behavioural practice/rehearsal	6	3	3	1.00
12. Graded tasks	4	2	2	1.00
13. Adding objects to the environment	2	1	1	1.00
14. Self-monitoring of behaviour	2	1	1	1.00
15. Demonstration of the behaviour	5	2	3	0.67
16. Self-monitoring of outcome of behaviour	1	1	0	0.00
17. Monitoring of behaviour by others without feedback	1	0	1	0.00
18. Information about emotional consequences	1	1	0	0.00
19. Review behaviour goal	1	1	0	0.00

Ratios of intervention promise to BCTs are presented in Table 5. Nine promising BCTs (ratios ≥2) were identified: action planning; social support; problem solving; goal setting behaviour; credible source; biofeedback; feedback on behaviour and information about health consequences.

## Discussion

Nine studies were identified that targeted free-living physical activity, of which 6 were classified as promising based on observed within- or between-group changes in outcome measures. None of the studies identified targeted sedentary behaviour. Six interventions were rated as very or quite promising, all of which had an element of supervised support that was tailored to characteristics of participants. All nine studies were rated as having a high or unclear risk of bias, which prohibited any conclusions about their potential for improving physical activity in stroke survivors.

### Intervention content

Interventions were limited by poor descriptions of the rationale behind the mode of delivery, form and content. Only two ‘quite promising’ interventions were developed with reference to a pre-specified model or theory of behaviour change, the Health Action Process Approach [45] and Social Cognitive Theory [46]. Limited use of theory is consistent with previous findings in the context of behavioural interventions for stroke [17]. Although a number of constructs of these theories appear to have been targeted by intervention components, poor fidelity assessment scores for these (and other promising interventions) highlight the possibility that they may not have been delivered as planned and impacted on observed effects.

Procedures and materials (“what”) and mode of delivery (“how”) varied across interventions. The most promising intervention used a supervised structured exercise programme incorporating aerobic exercises and strength training.

Three supervised structured exercise interventions used physical function and fitness as primary outcomes, assessing physical activity change as a secondary outcome. Although improving function and fitness through structured exercise may indirectly influence long-term free living physical activity behaviour. Although stroke survivors report high levels of satisfaction with group-based exercise programmes, a number of barriers exist to participation in these programmes, including cost, access and sustainable resources [48, 49]. The promising interventions used a number of other procedures, including supported self-management, which presents fewer barriers to participation after stroke and facilitate continuation beyond the intervention period.

Supported self-management incorporating BCTs such as goal setting, action planning and problem-solving delivered <one year post stroke) has been shown to improve extended activities of daily living [14] and this appears to be feasible for targeting physical activity after stroke [15, 16]. Indeed four of the six promising interventions utilised support self-management programmes. Two of the promising interventions were supported self-management programmes delivered in a single session. This could be an economical alternative to face to face structured exercise sessions where high costs and requirements for specialist training have been reported previously as barriers to implementation [50]. However, other promising interventions consisted of ≥24 contact points during intervention delivery, which may reduce the cost-effectiveness of these programmes, and work to establish the preferred intensity of contact should be undertaken as part of intervention design.

Two quite promising interventions were delivered by telephone, which could be more economical than face-to face delivery. None of the interventions were delivered remotely via the Internet; however a recent feasibility study has reported that this may be a feasible mode of delivery [15]. The mode of intervention delivery is driven by individual preference [51]. Preferences have been found to be different in stroke survivors and healthy controls, with stroke survivors reporting stronger preferences to exercise in a gym or fitness centre in a group-setting [52].

“Who” delivers an intervention is an important consideration of intervention design [53]. The current review did not identify “who’” the optimal type of interventionist, but rather that a range of healthcare professionals can deliver physical activity interventions to stroke survivors. The presence of a credible source of information (i.e., a healthcare professional) was identified as a promising BCT. Previous work has demonstrated that the credibility of the source is an important factor in the success of interventions [54–56].

It was not possible to determine from the review findings “when” the optimal time in the care pathway to deliver an intervention, include ‘where’ they should be delivered. Creating physical activity habits before patients potentially become deconditioned and in the early stages of rehabilitation [57], when recovery is maximal [58], is an important consideration. Initiating physical activity interventions in the early stages after stroke may increase the likelihood that a patient is given access to a health care professional who could deliver the intervention. Conversely, some individuals may feel overwhelmed in the acute stages after stroke and may wish to engage at a later time, in their own environment, when fatigue levels may have reduced and cognitive ability improved [59].

In terms of where the interventions are delivered, it appears that they can be successfully delivered across a range of settings.

“How well” the intervention was delivered, in terms of planning and actual delivery, was poorly described. Fidelity of intervention delivery is extremely important as efficacy can only be determined if an intervention has been delivered as intended [60]. In the very promising [31] and quite promising interventions [32–36] median treatment fidelity scores were < 50%. Scores were particularly poor for monitoring and improving provider training, monitoring and improving participant receipt of intervention and monitoring and improving participant enactment of intervention skills. In order for complex behavioural interventions to be effective, receipt and enactment should be measured to ensure participants have the skills to effectively self-manage their physical activity behaviour in the longer-term [61].

We identified nine promising BCTs that fell into five different groupings [20]: (i) Natural Consequences (information about health consequences [*written, verbal, visual information about health consequences of performing the behaviour*] and information about social and environmental consequences [*written, verbal, visual information about social and environmental consequences of performing the behaviour*]); (ii) Goals and Planning (goal setting-behaviour [*set/agree on a goal in terms of the behaviour to be achieved*], problem-solving [*analyse, or prompt the person to analyse, factors influencing the behaviour and generate/select strategies that include overcoming barriers and/or increasing facilitators]* and action planning [*prompt detailed planning of performance of the behaviour in terms of context, frequency, duration and/or intensity*]); (iii) Feedback and Monitoring (feedback on behaviour [*monitor and provide informative or evaluative feedback on performance of the behaviour such as form, frequency, duration, intensity*)] and biofeedback [*provide feedback about the physiological or biochemical state of the body using an external monitoring device*]); (iv) Social Support (social support unspecified [*advise on, arrange or provide social support from friends, relatives, colleagues, buddies or staff, or non-contingent praise or reward for performance of the behaviour*]); and (v) Comparison of Outcomes (credible source [*verbal or visual communication from a credible source in favour or against the behaviour; for example healthcare professionals*]).

### Outcome measures

None of the studies used the same outcome measure for assessing change in physical activity behaviour, and the use of subjective and objective measures prohibited a meta-analysis to enable a more accurate picture of the effectiveness of the interventions to be identified. There is a lack of consensus on the optimal measure with which to capture all the key elements of physical activity (e.g. intensity, frequency and duration) after stroke [62, 63] due to factors such as slow gait speed, hemiplegia and wheelchair use impacting on the accuracy of objective measurement via pedometers or accelerometers [64, 65]; and potential for social desirability and recall bias when using subjective measures of physical activity [66]. The use of subjective measures is particularly problematic with stroke survivors who have communication and cognitive problems. It would therefore be beneficial to standardise the use of outcome measures in this field to enable synthesis of future research findings and to establish intervention efficacy.

None of the studies retained for review involved interventions targeting sedentary behaviour. Sedentary behaviour is associated with increased cardiovascular disease incidence and mortality [2]. A better understanding of how to target the amount of time spent sedentary is vital as people with stroke are among the most sedentary. Breaking up sitting time may represent a more accessible intervention option for many stroke survivors [67]. At the time this review was conducted to our knowledge, only one study had been conducted specifically targeting sedentary behaviour after stroke [67]. This study was excluded from our review as it did not include follow-up measures of sedentary behaviour. Further studies assessing the effectiveness of interventions targeting sedentary behaviour after stroke with sedentary behaviour follow-up assessment measures (e.g. time spent sitting) are required.

### Strengths and limitations

A robust methodological approach and adherence to a published protocol and PRISMA are strengths of this review. The TIDieR framework and the BCT taxonomy allowed a thorough analysis of intervention components. This is a unique way of reporting systematic review findings. The robust assessment of an often neglected, but critically important issue of treatment fidelity is a further strength.

We grouped interventions according to promise, based on observed within- or between-group changes in outcome measures [28]. This method is useful where effect sizes cannot be determined. It also afforded the means by which BCTs can be linked to the apparent potential of an intervention to change behaviour (promise ratio). However, this method of determining potential is subjective in comparison to methods used in other reviews which determine potential based on standardised outcomes [28]. Intervention potential determined as “promise”, therefore, is less precise, meaning, for example, that interventions where changes in physical activity outcomes just reached statistical significance may have been grouped as “very promising” alongside interventions where changes reached a high level of significance. The use of promise/promise ratio also does not account for the size of the sample which may influence interpretation of results. Had it been possible to determine effect sizes, the determination of the promise ratios of BCTs would have been more precise.

Several interventions measured free-living physical activity as a secondary outcome. This limited the extent to which intervention components were described, such as any rationale or theory underpinning the intervention, fidelity of intervention delivery, and as such the extent to which conclusions could be made.

### Further research

Future research would benefit from establishing stroke survivor preferences for modes of delivery, setting and intensity (preferred number of contacts and session duration), including measurement of physical activity and sedentary behaviour. Interventions need to justify and utilise a theory/model of behaviour change and explore the optimal combination of promising BCTs within interventions. Further research on the development and impact of sedentary behaviour interventions after stroke are also warranted. The creation and maintenance of community/regional stroke registries to share data generated by research on interventions targeting physical activity and sedentary behaviour would further facilitate and progress this important field of research.

## Conclusions

Tailored interventions utilising nine promising BCTs within a supported self-management programme have potential to engage stroke survivors in physical activity behaviour change. However, limitations in intervention design, including sub-optimal fidelity assessment, and the lack of a standardised outcome measurement, prohibit any robust conclusions and highlight a need for further research in this area. Incorporation of the findings of this current review alongside in-depth qualitative work and an interactive co-design process (involving stroke survivors, their relatives and healthcare professionals) should be used to guide intervention development and to ultimately determine the most effective methods for influencing free-living physical activity and sedentary behaviour after stroke.

## Additional files

Additional file 1: Preferred Reporting Items for Systematic Reviews and Meta-Analyses (PRISMA) statement. (DOCX 29 kb)

Additional file 2: Example (MEDLINE) search strategy. (DOCX 12 kb)

Additional file 3: Example data extraction form. (DOCX 27 kb)

Additional file 4: Excluded Studies. (DOCX 21 kb)

## Abbreviations

BCTs: Behaviour change techniques
PRISMA: Preferred Reporting Items for Systematic Reviews and Meta-Analyses
TIDieR: Template for Intervention Description and Replication

## References

[1] Lee IM, Shiroma EJ, Lobelo F, Puska P, Blair SN, Katzmarzyk PT (2012). Lancet physical activity series working group. Effect of physical inactivity on major non-communicable diseases worldwide: an analysis of burden of disease and life expectancy. Lancet.

[2] Owen N, Healy GN, Matthews CE, Dunstan D (2010). Too much sitting: the population health science of sedentary behavior. Exerc Sport Sci Rev.

[3] Fini NA, Holland AE, Keating J, Simek J, Bernhardt J (2010). How physically active are people following stroke? Systematic review and quantitative synthesis. Phys Ther.

[4] Shaughnessy M, Michael KM, Sorkin JD, Macko RF (2005). Steps after stroke: capturing ambulatory recovery. Stroke.

[5] Moore SA, Hallsworth K, Ploetz T, Ford GA, Rochester L, Trenell MI (2013). Physical activity, sedentary behaviour and metabolic control following stroke: a cross-sectional and longitudinal study. PLoS One.

[6] Saunders DH, Sanderson M, Hayes S, Kilrane M, Greig CA, Brazzelli M, Mead GE. Physical fitness training for stroke patients. Cochrane Database Syst Rev. 2013:CD003316. 10.1002/14651858.CD003316.pub5. 10.1002/14651858.CD003316.pub6 PMC6464717 27010219

[7] Moore SA, Hallsworth K, Jakovljevic DG, Blamire AM, He J, Ford GA, Rochester L, Trenell MI (2015). Effects of community exercise therapy on metabolic, brain, physical and cognitive function following stroke: a randomised controlled pilot trial. Neurorehabil Neural Repair.

[8] Hackett ML, Yapa C, Parag V, Anderson CS (2005). Frequency of depression after stroke. A systematic review of observational studies. Stroke.

[9] Murray J, Young J, Forster A, Ashworth R (2003). Developing a primary care-based stroke model: the prevalence of longer-term problems experienced by patients and carers. Br J Gen Pract.

[10] Nicholson S, Sniehotta FF, van Wijck F, Greig CA, Johnston M, McMurdo ME, Dennis M, Mead GE (2013). A systematic review of perceived barriers and motivators to physical activity after stroke. Int J Stroke.

[11] Nicholson SL, Donaghy M, Johnston M, Sniehotta FF, van Wijck F, Johnston D, Greig C, McMurdo ME, Mead G (2014). A qualitative theory guided analysis of stroke survivors’ perceived barriers and facilitators to physical activity. Disabil Rehabil.

[12] Pang MYC, Eng JJ, Dawson AS, Gylfadottir S (2006). The use of aerobic exercise training in improving aerobic capacity in individuals with stroke: a meta-analysis. Clin Rehabil.

[13] Ivey F, Ryan AS, Hafer-Macko C, Goldberg A, Macko RF (2007). Treadmill aerobic training improves glucose tolerance and indices of insulin sensitivity. Stroke.

[14] Warner G, Packer T, Villeneuve M, Audulv A, Versnel J (2015). A systematic review of the effectiveness of stroke self-management programs for improving function and participation outcomes: self-management programs for stroke survivors. Disabil Rehabil.

[15] Jones TM, Dear BF, Hush JM, Titov N, Dean CM (2016). myMoves program: feasibility and acceptability study of a remotely delivered self-management program for increasing physical activity among adults with acquired brain injury living in the community. Phys Ther.

[16] Preston E, Dean CM, Ada L, Stanton R, Brauer S, Kuys S, Waddington G (2017). Promoting physical activity after stroke via self-management: a feasibility study. Top Stroke Rehabil.

[17] Morris JH, MacGillivray S, McFarlane S (2014). Interventions to promote long-term participation in physical activity after stroke: a systematic review of the literature. Arch Phys Med Rehabil.

[18] Craig P, Dieppe P, Macintyre S, Michie S, Nazareth I, Petticrew M (2013). Developing and evaluating complex interventions: the new Medical Research Council guidance. Int J Nurs Stud.

[19] Gourlan M, Bernard P, Bortolon C, Romain AJ, Lareyre O, Carayol M, Ninot G, Boiché J (2016). Efficacy of theory-based interventions to promote physical activity: a meta-analysis of randomised controlled trials. Health Psychol Rev.

[20] Michie S, Wood CE, Johnston M, Abraham C, Francis JJ, Hardeman W (2015). Behaviour change techniques: the development and evaluation of a taxonomic method for reporting and describing behaviour change interventions (a suite of five studies involving consensus methods, randomised controlled trials and analysis of qualitative data). Health Technol Assess.

[21] Avery L, Sniehotta F, Denton S, Steen N, McColl E, Taylor R, Trenell M (2014). Movement as medicine for type 2 diabetes: protocol for an open pilot study and external pilot clustered randomised controlled trial to assess acceptability, feasibility and fidelity of a multifaceted behavioural intervention targeting physical activity in primary care. Trials.

[22] Knittle K, De Gucht V, Hurkmans E, Peeters A, Ronday K, Maes S, Vlieland T (2013). Targeting motivation and self-regulation to increase physical activity among patients with rheumatoid arthritis: a randomised controlled trial. Clin Rheumatol.

[23] Hoffmann TC, Walker MF, Langhorne P, Eames S, Thomas E, Glasziou P (2015). What's in a name? The challenge of describing interventions in systematic reviews: analysis of a random sample of reviews of non-pharmacological stroke interventions. BMJ Open.

[24] Moore SA, Hrisos N, Avery L, Flynn D, Price C, Errington L. A systematic review assessing the effectiveness of interventions and component behaviour change strategies targeting long-term physical activity and/or sedentary behaviour in stroke survivors. PROSPERO. 2017:CRD42017059865 http://www.crd.york.ac.uk/PROSPERO/display_record.php?ID=CRD42017059865. Acccessed 21 May 2018.

[25] Moher D, Liberati A, Tetzlaff J, Altman DG (2009). Preferred reporting items for systematic reviews and meta-analyses: the PRISMA statement. J Clin Epidemiol.

[26] Michie S, Prestwich A (2010). Are interventions theory-based? Development of a theory coding scheme. Health Psychol.

[27] Higgins J, Green S. Cochrane handbook for systematic reviews of interventions. Oxford: Wiley; 2011.

[28] Gardner B, Smith L, Lorencatto F, Hamer M, Biddle SJH (2016). How to reduce sitting time? A review of behaviour change strategies used in sedentary behaviour reduction interventions among adults. Health Psychol Rev.

[29] Martin J, Chater A, Lorencatto F (2013). Effective behaviour change techniques in the prevention and management of childhood obesity. Int J Obes.

[30] Bellg AJ, Borrelli B, Resnick B, Hecht J, Minicucci DS, Ory M, Czajkowski S (2004). Enhancing treatment fidelity in health behavior change studies: best practices and recommendations from the NIH behavior change consortium. Health Psychol.

[31] Olney SJ, Nymark J, Brouwer B, Culham E, Day A, Heard J, Henderson M, Parvataneni K (2006). A randomized controlled trial of supervised versus unsupervised exercise programs for ambulatory stroke survivors. Stroke.

[32] Damush TM, Ofner S, Yu Z, Plue L, Nicholas G, Williams LS (2011). Implementation of a stroke self-management program. Transl Behav Med.

[33] Ludwig L, Kuderer B, Dettmers C (2016). Application of volitional training strategies in neurological rehabilitation to increase regular walking training - a pilot study. Neurol Rehabil.

[34] Morén C, Welmer AK, Hagströmer M, Karlsson E, Sommerfeld DK (2016). The effects of “physical activity on prescription” in persons with transient ischemic attack: a randomized controlled study. J Neurol Phys Ther.

[35] Severinsen K, Jakobsen JK, Pedersen AR, Overgaard K, Andersen H (2014). Effects of resistance training and aerobic training on ambulation in chronic stroke. Am J Phys Med Rehabil.

[36] Wan LH, You LM, Chen SX, Zhang XP, Mo MM, Zhang YM, Lu YW (2016). The effectiveness of a comprehensive reminder system in the secondary prevention of hypertensive ischaemic stroke: randomized controlled trial protocol. J Adv Nurs.

[37] Katz-Leurer M, Carmeli E, Shochina M (2003). The effect of early aerobic training on independence six months post stroke. Clin Rehabil.

[38] Mudge S, Barber PA, Stott NS (2009). Circuit-based rehabilitation improves gait endurance but not usual walking activity in chronic stroke: a randomized controlled trial. Arch Phys Med Rehabil.

[39] Sit JW, Yip VY, Ko SK, Gun AP, Lee JS (2007). A quasi-experimental study on a community-based stroke prevention programme for clients with minor stroke. J Clin Nurs.

[40] Keith RA, Granger CV, Hamilton BB, Sherwin FS (1987). The functional independence measure: a new tool for rehabilitation. Adv Clin Rehabil.

[41] Daughton DM, Fix AJ (1988). Human activity profile: professional manual.

[42] Aadahl M, JØrgensen T (2003). Validation of a new self-report instrument for measuring physical activity. Med Sci Sports Exerc.

[43] Lorig K, Stewart A, Ritter P, Gonzalez V, Laurent D, Lynch J. Outcome Measures for Health Education and Other Health Care Interventions. New York: Sage; 1996.

[44] Walker SN, Sechrist KR, Pender NJ (1987). The health-promoting lifestyle profile: development and psychometric characteristics. Nurs Res.

[45] Schwarzer R (1992). Self-efficacy in the adoption and maintenance of health behaviours: theoretical approaches and a new model.

[46] Bandura A (1986). Social foundations of thought and action: a social cognitive theory.

[47] Gearhart RF, Goss FL, Lagally KM, Jakicic JM, Gallagher J, Gallagher KI, Robertson RJ (2002). Ratings of perceived exertion in active muscle during high-intensity and low-intensity resistance exercise. J Strength Cond Res.

[48] Reed M, Harrington R, Duggan Á, Wood VA (2009). Meeting stroke survivors’ perceived needs: a qualitative study of a community-based exercise and education scheme. Clin Rehabil.

[49] Rimmer JH, Wang E, Smith D (2008). Barriers associated with exercise and community access for individuals with stroke. J Rehabil Res Dev.

[50] Mead G, Van Wijck F (2011). Physical fitness training after stroke: time to translate evidence to practice. J R Coll Physcians Edinb.

[51] Short CE, Vandelanotte C, Duncan MJ (2014). Individual characteristics associated with physical activity intervention delivery mode preferences among adults. Int J Behav Nutr Phys Act.

[52] Banks Geraldine, Bernhardt Julie, Churilov Leonid, Cumming Toby B. (2012). Exercise Preferences Are Different after Stroke. Stroke Research and Treatment.

[53] Luker J, Lynch E, Bernhardsson S, Bennett L, Bernhardt J (2015). Stroke survivors’ experiences of physical rehabilitation: a systematic review of qualitative studies. Arch Phys Med Rehabil.

[54] Parveen S, Islam MS, Begum M, Alam MU, Sazzad HM, Sultana R, Luby SP (2016). It’s not only what you say, it’s also how you say it: communicating nipah virus prevention messages during an outbreak in Bangladesh. BMC Public Health.

[55] Redfern J, Santo K, Coorey G, Thakkar J, Hackett M, Thiagalingam A, Chow CK (2016). Factors influencing engagement, perceived usefulness and behavioral mechanisms associated with a text message support program. PLoS One.

[56] Heron N, Kee F, Donnelly M, Cardwell C, Tully MA, Cupples ME (2016). Behaviour change techniques in home-based cardiac rehabilitation: a systematic review. Br J Gen Pract.

[57] Billinger SA, Arena R, Bernhardt J, Eng JJ, Franklin BA, Johnson CM, MacKay-Lyons M, Macko RF, Mead GE, Roth EJ, Shaughnessy M, Tang A (2014). Physical activity and exercise recommendations for stroke survivors: a statement for healthcare professionals from the American Heart Association/American Stroke Association. Stroke.

[58] Kwakkel G, Kollen B, Twisk J (2006). Impact of time on improvement of outcome after stroke. Stroke.

[59] Moore SA, Bednell L, Flynn D, Avery L (2018). An exploration of potential facilitators and barriers to change in long-term physical activity behavior in community dwelling stroke survivors.

[60] Borrelli B (2011). The assessment, monitoring, and enhancement of treatment fidelity in public health clinical trials. J Public Health Dent.

[61] Walton H, Spector A, Tombor I, Michie S (2017). Measures of fidelity of delivery of, and engagement with, complex, face-to-face health behaviour chnage interventions: a systematic review of measure quality. Br J Health Psychol.

[62] Kwakkel G, Lannin NA, Borschmann K, English C, Ali M, Churilov L, Saposnik G, Winstein C, van Wegen EEH, Wolf SL, Krakauer JW, Bernhardt J (2017). Standardized measurement of sensorimotor recovery in stroke trials: consensus-based core recommendations from the stroke recovery and rehabilitation roundtable. Neurorehabil Neural Repair.

[63] Fini NA, Holland AE, Keating J, Simek J, Bernhardt J (2015). How is physical activity monitored in people following stroke?. Disabil Rehabil.

[64] Carroll SL, Greig CA, Lewis SJ, McMurdo ME, Sniehotta FF, Johnston M, Johnston DW, Scopes J, Mead GE (2012). The use of pedometers in stroke survivors: are they feasible and how well do they detect steps?. Arch Phys Med Rehabil.

[65] Storm FA, Heller BW, Mazzà C (2015). Step detection and activity recognition accuracy of seven physical activity monitors. PLoS One.

[66] Rikli RE (2000). Reliability, validity and methodological issues in assessing physical activity in older adults. Res Q Exerc Sport.

[67] English C, Healy GM, Olds TS, Parfitt G, Borkoles E, Coates A, Kramer S, Bernhardt J (2015). Reducing sitting time after stroke: a phase ii safety and feasibility randomized controlled trial. Arch Phys Med Rehabil.

