# Supplementary material for: How should long-term free-living physical activity be targeted after stroke? A systematic review and narrative synthesis
Source: Int J Behav Nutr Phys Act. 2018 Oct 17;15:100. doi: 10.1186/s12966-018-0730-0 (PMC6192196; doi:10.1186/s12966-018-0730-0)
Supplement: Supplementary file 2 — Example (MEDLINE) search strategy. (DOCX 12 kb) [file 12966_2018_730_MOESM2_ESM.docx]

**Example (MEDLINE) search strategy**

Database: Ovid MEDLINE(R) without Revisions <1946 to February Week 1 2017>

Search Strategy:

1 exp Exercise/ (147874)

2 exercis$.mp. (278037)

3 exp Physical Fitness/ (24811)

4 fitness.mp. (60246)

5 physical$ activ$.mp. (69843)

6 exp Sports/ (151609)

7 sport$.mp. (70503)

8 Yoga/ (1965)

9 Tai Ji/ (780)

10 tai chi.mp. (943)

11 pilates.mp. (203)

12 bowling.mp. (325)

13 bowls.mp. (298)

14 Gardening/ (706)

15 Dancing/ (2283)

16 Sedentary Lifestyle/ (5176)

17 sedentary.mp. (21725)

18 seated.mp. (8260)

19 sitting.mp. (15698)

20 inactiv$.mp. (263451)

21 lying down.mp. (768)

22 or/1-21 (748830)

23 exp Stroke/ (102405)

24 cerebrovascular accident.mp. (3307)

25 Ischemic Attack, Transient/ (18780)

26 transient ischaemic attack.mp. (1294)

27 acquired brain injury.mp. (1192)

28 or/23-27 (119391)

29 randomized controlled trial.pt. (427876)

30 controlled clinical trial.pt. (91195)

31 randomized.ab. (324717)

32 placebo.ab. (161864)

33 drug therapy.fs. (1899234)

34 randomly.ab. (229220)

35 trial.ab. (334839)

36 groups.ab. (1440074)

37 or/29-36 (3625275)

38 exp animals/ not humans.sh. (4294918)

39 37 not 38 (3089173)

40 22 and 28 and 39 (1549)
