# Supplementary material for: How should long-term free-living physical activity be targeted after stroke? A systematic review and narrative synthesis
Source: Int J Behav Nutr Phys Act. 2018 Oct 17;15:100. doi: 10.1186/s12966-018-0730-0 (PMC6192196; doi:10.1186/s12966-018-0730-0)
Supplement: Supplementary file 3 — Example data extraction form. (DOCX 27 kb) [file 12966_2018_730_MOESM3_ESM.docx]

**Example data extraction form**

**General Information**

| **Date of data extraction** |  |
| --- | --- |
| **Data extracted by** |  |
| **Study ID** |  |
| **Corresponding author email** |  |

**Study Characteristics**

| **Aims / objectives** |  | | |
| --- | --- | --- | --- |
| **Study design** |  | | |
| **Number of study arms** |  | | |
| **Inclusion criteria** |  | | |
| **Exclusion criteria** |  | | |
| **Number of participants** | **Total sample size** | **Intervention group** | **Control/ Usual Care** |
|  |  |  |  |
| **Follow-up time period** |  | | |
| **Loss to follow-up** |  | | |
| **Brief details of setting**  (Country, inpatient/ESD/community setting) |  | | |

**Participant Characteristics**

| **Group**  **Demographics** | **Intervention group 1** | **Intervention group 2** | **Control / usual care Group** | **Overall** |
| --- | --- | --- | --- | --- |
| **Age**  (Mean/median/SD/IQR/range/not reported) |  |  |  |  |
| **Gender**  (Number/percentage/not reported) |  |  |  |  |
| **Group**  **Stroke Characteristics** | **Intervention group 1** | **Intervention group 2** | **Control / usual care Group** | **Overall** |
| **Type of stroke** (Infarct/haemorrhage/TACI/PACI/POCI/LACI) |  |  |  |  |
| **Length of time since stroke** (Mean/median/SD/IQR)/not reported) |  |  |  |  |
| **Functional level**  (Measured by Barthel, NIHSS, Motor assessment scale, etc.) |  |  |  |  |
| **Walking aid** (Y/N) |  |  |  |  |
| **Cognitive status** (MMSE, etc.) |  |  |  |  |
| **Depression score** (HAD, etc.) |  |  |  |  |

**Intervention Details**

| **Mode of delivery** | Attendance at one-to-one session with interventionist  Yes | | Attendance at exercise / PA class  Yes / No | Self-help information materials  Yes / No | Information / support via website:  Yes / No | Information / support via telephone:  Yes / No |
| --- | --- | --- | --- | --- | --- | --- |
| **Is there a supervised component of the intervention** (i.e. participant received supervision by study personnel/clinicians during physical activity/exercise)? | | | |  | | |
| **Interventionist** | |  | | Interventionist trained (/received training) in behaviour change? Yes / No If yes – please state: | | |
| **Duration of Intervention** | |  | | | | |
| **Type of exercise / PA targeted** | |  | | | | |
| **Description of intervention** | |  | | **Degree of personalisation:** | | |
| **Details of control / usual care intervention(s)** | |  | | **Degree of personalisation:** | | |
| **Explicit reference to theory** | | Yes / No | |  | | |

**Outcomes**

| **Outcomes** | **Description (including details of data collection method[s])** |
| --- | --- |
| Primary outcome (if stated) |  |
| Direct change in physical activity/exercise/sedentary behaviour |  |
| Indirect change in physical activity/sedentary behaviour (e.g. Frenchay Activities Index) |  |
| Other (please state) |  |

**Results**

**Outcome:**

|  | **Intervention Group 1**  Summary Statistics | | | | | **Intervention Group 2**  Summary Statistics | | | | | **Control/Usual Care**  Summary Statics | | | | |
| --- | --- | --- | --- | --- | --- | --- | --- | --- | --- | --- | --- | --- | --- | --- | --- |
|  |  |  |  |  |  |  |  |  |  |  |  |  |  |  |  |
| Mean (SD) |  |  |  |  |  |  |  |  |  |  |  |  |  |  |  |
| Median (IQR) |  |  |  |  |  |  |  |  |  |  |  |  |  |  |  |
| F (%) |  |  |  |  |  |  |  |  |  |  |  |  |  |  |  |
| **Details** (e.g., mean difference and 95% CIs): | | | | | | | | | | | | | | | |

**Outcome:**

|  | **Intervention Group 1**  Summary Statistics | | | | | **Intervention Group 2**  Summary Statistics | | | | | **Control/Usual Care**  Summary Statics | | | | | |
| --- | --- | --- | --- | --- | --- | --- | --- | --- | --- | --- | --- | --- | --- | --- | --- | --- |
|  |  |  |  |  |  |  |  |  |  |  |  |  |  |  |  |  |
| Mean (SD) |  |  |  |  |  |  |  |  |  |  |  |  |  |  |  |  |
| Median (IQR) |  |  |  |  |  |  |  |  |  |  |  |  |  |  |  |  |
| F (%) |  |  |  |  |  |  |  |  |  |  |  |  |  |  |  |  |
| **Details** (e.g., mean difference and 95% CIs): | | | | | | | | | | | | | | | | |

**Methodological Quality^1^**

| **Domain** | **Review author’s judgement**  (high / low / unclear risk of bias) | **Support for judgement** |
| --- | --- | --- |
| *Selection bias.* | | |
| **Random sequence generation.** |  |  |
| **Allocation concealment.** |  |  |
| *Performance bias.* | | |
| **Blinding of participants** *Assessments should be made for each main outcome (or class of outcomes).* |  |  |
| **Blinding of personnel** *Assessments should be made for each main outcome (or class of outcomes).* |  |  |
| *Detection bias.* | | |
| **Blinding of outcome assessment** *Assessments should be made for each main outcome (or class of outcomes)*. |  |  |
| *Attrition bias.* | | |
| **Incomplete outcome data** *Assessments should be made for each main outcome (or class of outcomes).* |  |  |
| *Reporting bias.* | | |
| **Selective reporting.** |  |  |
| *Other bias.* | | |
| **Other sources of bias.** |  |  |

^1.^ Higgins, J. P., & Green, S. (Eds.). (2011). Cochrane handbook for systematic reviews of interventions (Vol. 4). John Wiley & Sons.

| **Theory** | | **Please state details** |
| --- | --- | --- |
| Was a theory used? | Yes No Unsure |  |
| Which theory was used? |  |  |
| Were all constructs targeted by the intervention? | Yes No Unsure |  |
| Were changes in each construct measured? | Yes No Unsure |  |

**Theoretical Basis**

**Primary Behaviour Change Aim**

| To increase physical activity |  |
| --- | --- |
| To reduce sedentary behaviour |  |
| Joint: To increase physical activity and reduce sedentary behaviour |  |
| Other (please state) |  |

**Treatment Fidelity Assessment**

| **Does the paper report any treatment fidelity measures?**  (Bellg et al., 2004) |  | **Details** |
| --- | --- | --- |
| **1) Treatment fidelity strategies for design of study** |  |  |
| - Ensure same treatment dose within conditions | Yes / No |  |
| - Ensure equivalent dose across conditions | Yes / No |  |
| - Plan for implementation setbacks | Yes / No |  |
| **2) Treatment fidelity strategies for monitoring and improving provider training** |  |  |
| - Standardize training | Yes / No |  |
| - Ensure provider skill acquisition | Yes / No |  |
| - Minimize “drift” in provider skills | Yes / No |  |
| - Accommodate provider differences | Yes / No |  |
| **3) Treatment fidelity strategies for monitoring and improving delivery of treatment** |  |  |
| - Control for provider differences | Yes / No |  |
| - Reduce differences within treatment | Yes / No |  |
| - Ensure adherence to treatment protocol | Yes / No |  |
| - Minimize contamination between conditions | Yes / No |  |
| **4) Treatment fidelity strategies for monitoring and improving receipt of treatment** |  |  |
| - Ensure participant comprehension | Yes / No |  |
| - Ensure participant ability to use cognitive skills | Yes / No |  |
| - Ensure participant ability to perform behavioural skills | Yes / No |  |
| **5) Treatment fidelity strategies for monitoring and improving enactment of treatment skills** |  |  |
| - Ensure participant use of cognitive skills | Yes / No |  |
| - Ensure participant use of behavioural skills | Yes / No |  |

**TIDieR (Template for Intervention Description and Replication) Checklist**

| **Item** | **Description** | **Where located**  (page no/other) |
| --- | --- | --- |
| **Brief Name:** Provide the name or a phrase that describes the intervention. |  |  |
| **Why:** Describe any rationale, theory or goal of the elements essential to the intervention. |  |  |
| **What** (Materials): Describe any physical or information materials used in the intervention, including those provided to participants or used in intervention delivery or in training of intervention providers. Provide information on where the materials can be accessed |  |  |
| **What** (Procedures): Describe each of the procedures, activities, and/or processes used in the intervention, including any enabling or support activities. |  |  |
| **Who Provided:** For each category of intervention provider, describe their expertise, background and any specific training given. |  |  |
| **How:** Describe the modes of delivery (e.g. face-to-face or by some other mechanism, such as internet or telephone) of the intervention and whether it was provided individually or in a group. |  |  |
| **Where:** Describe the type(s) and location(s) where the intervention occurred, including any necessary infrastructure or relevant features. |  |  |
| **When And How Much:** Describe the number of times the intervention was delivered and over what period of time including the number of sessions, their schedule, and their duration, intensity or dose. |  |  |
| **Tailoring:** If the intervention was planned to be personalised, titrated or adapted, then describe what, why, when, and how. |  |  |
| **Modifications:** If the intervention was modified during the course of the study, describe the changes (what, why, when, and how). |  |  |
| **How Well:** Planned: if intervention adherence or fidelity was assessed, describe how and by whom, and if any strategies were used to maintain or improve fidelity, describe them. |  |  |
| **How Well:** Actual: If intervention adherence or fidelity was assessed, describe the extent to which the intervention was delivered as planned. |  |  |

**BCTs**

| **Technique** | **Yes (describe with page numbers in paper)** | **No** | **Unsure** |
| --- | --- | --- | --- |
| 1.1 Goal setting (behaviour) |  |  |  |
| 1.2 Problem solving |  |  |  |
| 1.3 Goal setting (outcome) |  |  |  |
| 1.4 Action planning |  |  |  |
| 1.5 Review behaviour goal(s) |  |  |  |
| 1.6 Discrepancy between current behaviour and goal |  |  |  |
| 1.7 Review outcome goal(s) |  |  |  |
| 1.8 Behavioural contract |  |  |  |
| 1.9 Commitment |  |  |  |
| 2.1 Monitoring of behaviour by others without feedback |  |  |  |
| 2.2 Feedback on behaviour |  |  |  |
| 2.3 Self-monitoring of behaviour |  |  |  |
| 2.4 Self-monitoring of outcome(s) of behaviour |  |  |  |
| 2.5 Monitoring outcome(s) of behaviour by others without feedback |  |  |  |
| 2.6 Biofeedback |  |  |  |
| 2.7 Feedback on outcome(s) of behaviour |  |  |  |
| 3.1 Social support (unspecified) |  |  |  |
| 3.2 Social support (practical) |  |  |  |
| 3.3 Social support (emotional) |  |  |  |
| 4.1 Instruction on how to perform a behaviour |  |  |  |
| 4.2 Information about antecedents |  |  |  |
| 4.3 Re-attribution |  |  |  |
| 4.4 Behavioural experiments |  |  |  |
| 5.1 Information about health consequences |  |  |  |
| 5.2 Salience of consequences |  |  |  |
| 5.3 Information about social and environmental consequences |  |  |  |
| 5.4 Monitoring of emotional consequences |  |  |  |
| 5.5 Anticipated regret |  |  |  |
| 5.6 Information about emotional consequences |  |  |  |
| 6.1 Demonstration of the behaviour |  |  |  |
| 6.2 Social comparison |  |  |  |
| 6.3 Information about others' approval |  |  |  |
| 7.1 Prompts/cues |  |  |  |
| 7.2 Cue signalling reward |  |  |  |
| 7.3 Reduce prompts/cues |  |  |  |
| 7.4 Remove access to the reward |  |  |  |
| 7.5 Remove aversive stimulus |  |  |  |
| 7.6 Satiation |  |  |  |
| 7.7 Exposure |  |  |  |
| 7.8 Associative learning |  |  |  |
| 8.1 Behavioural practice/ rehearsal |  |  |  |
| 8.2 Behaviour substitution |  |  |  |
| 8.3 Habit formation |  |  |  |
| 8.4 Habit reversal |  |  |  |
| 8.5 Overcorrection |  |  |  |
| 8.6 Generalisation of a target behaviour |  |  |  |
| 8.7 Graded tasks |  |  |  |
| 9.1 Credible source |  |  |  |
| 9.2 Pros and cons |  |  |  |
| 9.3 Comparative imagining of future outcomes |  |  |  |
| 10.1 Material incentive (behaviour) |  |  |  |
| 10.2 Material reward (behaviour) |  |  |  |
| 10.3 Non-specific reward |  |  |  |
| 10.4 Social reward |  |  |  |
| 10.5 Social incentive |  |  |  |
| 10.6 Non-specific incentive |  |  |  |
| 10.7 Self-incentive |  |  |  |
| 10.8 Incentive (outcome) |  |  |  |
| 10.9 Self-reward |  |  |  |
| 10.10 Reward (outcome) |  |  |  |
| 10.11 Future punishment |  |  |  |
| 11.1 Pharmacological support |  |  |  |
| 11.2 Reduce negative emotions |  |  |  |
| 11.3 Conserving mental resources |  |  |  |
| 11.4 Paradoxical instructions |  |  |  |
| 12.1 Restructuring the physical environment |  |  |  |
| 12.2 Restructuring the social environment |  |  |  |
| 12.3 Avoidance/reducing exposure to cues for the behaviour |  |  |  |
| 12.4 Distraction |  |  |  |
| 12.5 Adding objects to the environment |  |  |  |
| 12.6 Body changes |  |  |  |
| 13.1 Identification of self as role model |  |  |  |
| 13.2 Framing/reframing |  |  |  |
| 13.3 Incompatible beliefs |  |  |  |
| 13.4 Valued self-identity |  |  |  |
| 13.5 Identity associated with changed behaviour |  |  |  |
| 14.1 Behaviour cost |  |  |  |
| 14.2 Punishment |  |  |  |
| 14.3 Remove reward |  |  |  |
| 14.4 Reward approximation |  |  |  |
| 14.5 Rewarding completion |  |  |  |
| 14.6 Situation-specific reward |  |  |  |
| 14.7 Reward incompatible behaviour |  |  |  |
| 14.8 Reward alternative behaviour |  |  |  |
| 14.9 Reduce reward frequency |  |  |  |
| 14.10 Remove punishment |  |  |  |
| 15.1 Verbal persuasion about capability |  |  |  |
| 15.2 Mental rehearsal of successful performance |  |  |  |
| 15.3 Focus on past success |  |  |  |
| 15.4 Self-talk |  |  |  |
| 16.1 Imaginary punishment |  |  |  |
| 16.2 Imaginary reward |  |  |  |
| 16.3 Vicarious consequences |  |  |  |
