# Supplementary material for: How should long-term free-living physical activity be targeted after stroke? A systematic review and narrative synthesis
Source: Int J Behav Nutr Phys Act. 2018 Oct 17;15:100. doi: 10.1186/s12966-018-0730-0 (PMC6192196; doi:10.1186/s12966-018-0730-0)
Supplement: Supplementary file 4 — Excluded Studies. (DOCX 21 kb) [file 12966_2018_730_MOESM4_ESM.docx]

**Excluded Studies**

Batcho, C. S., Stoquart, G., & Thonnard, J. L. (2013). BRISK WALKING CAN PROMOTE FUNCTIONAL RECOVERY IN CHRONIC STROKE PATIENTS. *Journal of Rehabilitation Medicine, 45*(9), 854-859. doi:10.2340/16501977-1211

Boss, H. M., Van Schaik, S. M., Deijle, I. A., De Melker, E. C., Van Den Berg, B. T. J., Bosboom, W. M. J., . . . Van Den Berg-Vos, R. M. (2013). Motives & move it-post-stroke care including physical exercise; a one-year follow-up pilot study. *Cerebrovascular Diseases, 35*, 738.

Bower, K. J., Louie, J., Landesrocha, Y., Seedy, P., Gorelik, A., & Bernhardt, J. (2015). Clinical feasibility of interactive motion-controlled games for stroke rehabilitation. *Journal of Neuroengineering & Rehabilitation, 12*, 63.

Boysen, G., Krarup, L., Zeng, X., Oskedra, A., Korv, J., Andersen, G., . . . Truelsen, T. (2009). ExStroke Pilot Trial of the effect of repeated instructions to improve physical activity after ischaemic stroke: a multinational randomised controlled clinical trial. *BMJ: British Medical Journal (Overseas & Retired Doctors Edition), 339*(7715), b2810-b2810. doi:10.1136/bmj.b2810

Cadilhac, D. A., Hoffmann, S., Kilkenny, M., Lindley, R., Lalor, E., Osborne, R. H., & Batterbsy, M. (2011). A phase II multicentered, single-blind, randomized, controlled trial of the stroke self-management program. *Stroke, 42*(6), 1673-1679.

Calugi, S., Taricco, M., Rucci, P., Fugazzaro, S., Stuart, M., Dallolio, L., . . . investigators, E. F. G. (2016). Effectiveness of adaptive physical activity combined with therapeutic patient education in stroke survivors at twelve months: a non-randomized parallel group study. *European journal of physical & rehabilitation medicine., 52*(1), 72-80.

Clanchy, K. M., Tweedy, S. M., & Trost, S. G. (2016). Evaluation of a Physical Activity Intervention for Adults with Brain Impairment: A Controlled Clinical Trial. *Neurorehabilitation and Neural Repair, 30*(9), 854-865.

Combs-Miller, S. A., Kalpathi Parameswaran, A., Colburn, D., Ertel, T., Harmeyer, A., Tucker, L., & Schmid, A. A. (2010). Body weight-supported treadmill training vs. overground walking training for persons with chronic stroke: a pilot randomized controlled trial. *Clinical Rehabilitation, 24*(9), 873-884. doi:10.1177/0269215514520773

Dean, C., Rissel, C., Sharkey, M., Sherrington, C., Cumming, R., Lord, S., . . . Barker, R. (2010). Ongoing exercise opportunities to prevent falls and enhance mobility in community dwellers after stroke: The stroke club trial. *International Journal of Stroke, 5*, 12.

Dean, C., Sherrington, C., Rissel, C., Sharkey, M., Cumming, R., Lord, S., . . . Barker, R. (2011). Exercise intervention to prevent falls, enhance mobility and increase physical activity in community dwellers after stroke: The stroke club trial. *Physiotherapy (United Kingdom), 97*, eS274-eS275.

Dean, C. M., Ada, L., & Lindley, R. I. (2014). Treadmill training provides greater benefit to the subgroup of community-dwelling people after stroke who walk faster than 0.4m/s: a randomised trial. *Journal of Physiotherapy, 60*(2), 97-101.

Dean, C. M., Rissel, C., Sherrington, C., Sharkey, M., Cumming, R. G., Lord, S. R., . . . O’Rourke, S. (2012). Exercise to Enhance Mobility and Prevent Falls After Stroke: The Community Stroke Club Randomized Trial. *Neurorehabilitation & Neural Repair, 26*(9), 1046-1057. doi:10.1177/1545968312441711

Dorsch, A., Thomas, S., Xu, C., Kaiser, W., & Dobkin, B. (2014). Implementation of a multicenter, international, randomized clinical trial in subacute stroke patients using wireless health technology. *Neurorehabilitation and Neural Repair, 28 (4)*, NP17.

Duncan, P. W., Sullivan, K. J., Behrman, A. L., Azen, S. P., Wu, S. S., Nadeau, S. E., . . . Tilson, J. K. (2011). Body-weight-supported treadmill rehabilitation after stroke. *New England Journal of Medicine, 364*(21), 2026-2036. doi:10.1056/NEJMoa1010790

English, C., Coates, A., Olds, T., Healy, G., Parfitt, G., Borkoles, E., & Bernhardt, J. (2015). 'Sit less, move more': A phase II safety and feasibility trial. *International Journal of Stroke, 10*, 33-34.

English, C., Healy, G. N., Olds, T., Parfitt, G., Borkoles, E., Coates, A., . . . Bernhardt, J. (2016). Reducing Sitting Time After Stroke: A Phase II Safety and Feasibility Randomized Controlled Trial. *Archives of Physical Medicine & Rehabilitation, 97*(2), 273-280.

Faulkner, J., Lambrick, D., Woolley, B., Stoner, L., Wong, L. K., & McGonigal, G. (2012). Health-enhancing physical activity programme (HEPAP) for transient ischaemic attack and non-disabling stroke: Recruitment and compliance. *New Zealand Medical Journal, 125*(1364).

Faulkner, J., Lambrick, D., Woolley, B., Stoner, L., Wong, L. K., & McGonigal, G. (2013). Effects of early exercise engagement on vascular risk in patients with transient ischemic attack and nondisabling stroke. *Journal of Stroke & Cerebrovascular Diseases, 22*(8), e388-396.

Faulkner, J., Lambrick, D., Woolley, B., Stoner, L., Wong, L. K., & McGonigal, G. (2014). The long-term effect of exercise on vascular risk factors and aerobic fitness in those with transient ischaemic attack: a randomized controlled trial. *Journal of Hypertension, 32*(10), 2064-2070.

Faulkner, J., Stoner, L., Lanford, J., Jolliffe, E., Mitchelmore, A., & Lambrick, D. (2016). Long-Term Effect of Participation in an Early Exercise and Education Program on Clinical Outcomes and Cost Implications, in Patients with TIA and Minor, Non-Disabling Stroke. *Translational Stroke Research*, 1-8.

Flansbjer, U. B., Brogardh, C., & Lexell, J. (2011). Long term effects of progressive resistance training after stroke: A 4-year follow-up. *Physiotherapy (United Kingdom), 97*, eS346.

Fleck, R. J., Richardson, J., Hladysh, G., McBay, C., Elizabeth, M., Thorlakson, R., . . . LeBlanc, K. (2012). FIT for FUNCTION community stroke wellness program: A pilot randomized controlled trial. *Stroke, 43 (11)*, e120.

Fritz, S., Peters, D., Merlo, A., & Donley, J. (2013). Active video-gaming effects on balance and mobility in individuals with chronic stroke: A randomized controlled trial. *Topics in Stroke Rehabilitation, 20*(3), 218-225.

Galvin, R., Cusack, T., O'Grady, E., Murphy, B., & Stokes, E. (2011). Family mediated exercise intervention [fame]: Evaluation of a novel form of exercise delivery after stroke. *Physiotherapy (United Kingdom), 97*, eS387-eS388.

Gill, L., & Sullivan, K. A. (2011). Boosting exercise beliefs and motivation through a psychological intervention designed for poststroke populations. *Topics in Stroke Rehabilitation, 18*(5), 470-480.

Gillham, S., & Endacott, R. (2010). Impact of enhanced secondary prevention on health behaviour in patients following minor stroke and transient ischaemic attack: a randomized controlled trial. *Clinical Rehabilitation, 24*(9), 822-830.

Gillham, S. D. (2009). Does enhanced secondary prevention intervention after minor stroke and Transient Ischaemic Attack (TIA) affect readiness to change health behaviour? *International Journal of Stroke, 4*, 27.

Giraux, P., Raffin, E., & Pl, M. R. (2012). Efficacy of long term physical therapy on walking activity in chronic stroke: Interim analysis, Impact de la kinesitherapie d'entretien sur l'activite de marche des patients hemiplegiques: resultats intermediaires. [French, English]. *Annals of Physical and Rehabilitation Medicine, 55*, e11+e13-e14.

Green, J., Forster, A., Bogle, S., & Young, J. (2002). Physiotherapy for patients with mobility problems more than 1 year after stroke: a randomised controlled trial. *Lancet, 359*(9302), 199-203.

Green, T., Haley, E., Eliasziw, M., & Hoyte, K. (2007). Education in stroke prevention: efficacy of an educational counselling intervention to increase knowledge in stroke survivors. *Canadian Journal of Neuroscience Nursing, 29*(2), 13-20.

Gunnes, M., Indredavik, B., & Askim, T. (2015). A prospective longitudinal study assessing stroke patients' adherence to a long-term follow-up program applied in a randomized controlled trial. *International Journal of Stroke, 10*, 165.

Harrington, R., Taylor, G., Hollinghurst, S., Reed, M., Kay, H., & Wood, V. A. (2010). A community-based exercise and education scheme for stroke survivors: a randomized controlled trial and economic evaluation. *Clinical Rehabilitation, 24*(1), 3-15.

Hesse, S., Eich, H. J., Mach, H., Parchmann, H., & Werner, C. (2005). Aerobic treadmill training plus physiotherapy improves walking speed and capacity in subacute, moderately affected patients after stroke. *Neurologie und Rehabilitation, 11*(1), 7-12.

Hill, V. A., & Towfighi, A. (2014). Healthy eating and lifestyle after stroke: A pilot lifestyle intervention. *Archives of Physical Medicine and Rehabilitation, 95 (10)*, e30-e31.

Hui-Chan, C. W., Ng, S. S., & Mak, M. K. (2009). Effectiveness of a home-based rehabilitation programme on lower limb functions after stroke. *Hong Kong Medical Journal, 15*(3 Suppl 4), 42-46.

Huijbregts, M. P. J., Myers, A. M., Streiner, D., & Teasell, R. (2008). Implementation, Process, and Preliminary Outcome Evaluation of Two Community Programs for Persons with Stroke and Their Care Partners. *Topics in Stroke Rehabilitation, 15*(5), 503-520. doi:10.1310/tsr1505-503

Immink, M. A., Chan, W. L., & Hillier, S. (2012). A pilot randomized controlled trial of yoga therapy supplemented exercise interventions for motor function and quality of life outcomes in patients with chronic post-stroke hemiparesis. *Journal of Sport & Exercise Psychology, 34*, S91-S91.

Jones, T. M., Dear, B. F., Hush, J. M., Titov, N., & Dean, C. M. (2016). myMoves Program: Feasibility and Acceptability Study of a Remotely Delivered Self-Management Program for Increasing Physical Activity Among Adults With Acquired Brain Injury Living in the Community. *Physical Therapy, 96*(12), 1982-1993.

Krarup, L. H., Lindahl, M., Truelsen, T., Gluud, C., & Boysen, G. (2010). The risk of falling after stroke is associated with physical inactivity. *Cerebrovascular Diseases, 29*, 72.

Kumaran, D. S., Rao, B. K., Rao, S. N., & Kamath, A. (2016). EFFECT OF A TASK AND CONTEXT BASED EXERCISE PROGRAM (TCEP) ON IMPROVING WALKING FUNCTION IN COMMUNITY DWELLING STROKE SURVIVORS: A RANDOMIZED CONTROLLED TRIAL. *International Journal of Stroke, 11*(SUPP 3), 59-59.

Kwok, C. M., Lui, H. T., Hui, K. F., Wong, K. Y., Lam, H. Y., & Yick, C. Y. (2012). Intensify secondary stroke prevention with quality of life wellbeing and functions restoration in the active lifestyle therapeutic exercise program. *Cerebrovascular Diseases, 34*, 135.

Langhammer, B., Lindmark, B., & Stanghelle, J. (2014). Are effects of a 1-year long-term intervention period in persons with stroke sustained 3 years after? The longitudinal follow-up of a randomized controlled trial. *Brain Injury, 28 (5-6)*, 556.

Langhammer, B., Lindmark, B., & Stanghelle, J. K. (2014). Physiotherapy and physical functioning post-stroke: exercise habits and functioning 4 years later? Long-term follow-up after a 1-year long-term intervention period: a randomized controlled trial. *Brain Injury, 28*(11), 1396-1405.

Logan, P. A., Armstrong, S., Avery, T. J., Barer, D., Barton, G. R., Darby, J., . . . Leighton, M. P. (2014). Rehabilitation aimed at improving outdoor mobility for people after stroke: a multicentre randomised controlled study (the Getting out of the House Study). *Health Technology Assessment, 18*(8), 1-114. doi:10.3310/hta18290

Lorig, K., Ritter, P. L., Plant, K., Laurent, D. D., Kelly, P., & Rowe, S. (2013). The South Australia health chronic disease self-management Internet trial. *Health Education & Behavior, 40*(1), 67-77.

Maguire, C., Sieben, J. M., Erzer, F., Goepfert, B., Frank, M., Ferber, G., . . . de Bie, R. A. (2012). How to improve walking, balance and social participation following stroke: A comparison of the long term effects of two walking aids--canes and an orthosis TheraTogs--on the recovery of gait following acute stroke. A study protocol for a multi-centre, single blind, randomised control trial. *BMC Neurology, 12*. doi:10.1186/1471-2377-12-18

Malagoni, A. M., Cavazza, S., Ferraresi, G., Grassi, G., Felisatti, M., Lamberti, N., . . . Manfredini, F. (2016). Effects of a "test in-train out" walking program versus supervised standard rehabilitation in chronic stroke patients: A feasibility and pilot randomized study. *European journal of physical and rehabilitation medicine, 52*(3), 279-287.

McCluskey, A., Ada, L., Kelly, P. J., Middleton, S., Goodall, S., Grimshaw, J. M., . . . Karageorge, A. (2016). A behavior change program to increase outings delivered during therapy to stroke survivors by community rehabilitation teams: The Out-and-About trial. *International Journal of Stroke, 11*(4), 425-437. doi:10.1177/1747493016632246

Morgenstern, L., Conley, K., Sanchez, B., Sais, E., & Brown, D. (2015). Behavior change goals in primary stroke prevention: The stroke health and risk education (share) project. *International Journal of Stroke, 10*, 16.

Pang, M. Y., Ashe, M. C., Eng, J. J., McKay, H. A., & Dawson, A. S. (2006). A 19-week exercise program for people with chronic stroke enhances bone geometry at the tibia: a peripheral quantitative computed tomography study. *Osteoporosis International, 17*(11), 1615-1625.

Park, H. J., Oh, D. W., Kim, S. Y., & Choi, J. D. (2011). Effectiveness of community-based ambulation training for walking function of post-stroke hemiparesis: a randomized controlled pilot trial. *Clinical Rehabilitation, 25*(5), 451-459.

Preston, E., Dean, C., Ada, L., Stanton, R., & Waddington, G. (2014). Promoting physical activity after stroke via self management: Protocol for a feasibility study. *International Journal of Stroke, 9*, 32.

Richardson, J., Fleck, R., Hladysh, G., McBay, C., MacKay, E., Thorlakson, R., . . . Le Blanc, K. (2011). Fit for function: A community wellness program for persons with stroke. *Stroke, 42 (11)*, e596.

Sandberg, K., Kleist, M., Falk, L., & Enthoven, P. (2016). Effects of Twice-Weekly Intense Aerobic Exercise in Early Subacute Stroke: A Randomized Controlled Trial. *Archives of Physical Medicine and Rehabilitation, 97*(8), 1244-1253.

Schonberger, M., Hansen, N. R., Pedersen, D. T., Zeeman, P., & Jorgensen, J. R. (2010). The relationship between physical fitness and work integration following stroke. *Brain Impairment, 11*(3), 262-269.

Shaughnessy, M., & Resnick, B. M. (2009). Using theory to develop an exercise intervention for patients post stroke. *Topics in Stroke Rehabilitation, 16*(2), 140-146.

Shaughnessy, M., & Stookey, A. (2012). Reshaping exercise habits and beliefs (REHAB): A randomized trial of home-based exercise in sub-acute stroke. *Stroke. Conference, 43*(2 Meeting Abstracts).

Stuart, M., Benvenuti, F., Macko, R., Taviani, A., Segenni, L., Mayer, F., . . . Weinrich, M. (2009). Community-Based Adaptive Physical Activity Program for Chronic Stroke: Feasibility, Safety, and Efficacy of the Empoli Model. *Neurorehabilitation and Neural Repair, 23*(7), 726-734. doi:10.1177/1545968309332734

Taylor-Piliae, R. E., & Coull, B. M. (2012). Community-based Yang-style Tai Chi is safe and feasible in chronic stroke: a pilot study. *Clinical Rehabilitation, 26*(2), 121-131.

Tilson, J. K., Duncan, P. W., Wu, S. S., Cen, S. Y., Feng, Q., Sullivan, K. J., . . . Azen, S. P. (2011). Characterizing and identifying risk for falls in the leaps study: A randomized clinical trial. *Stroke, 42 (11)*, e621.

Tiozzo, E., Youbi, M., Dave, K., Perez-Pinzon, M., Rundek, T., Sacco, R. L., . . . Wright, C. B. (2015). Aerobic, Resistance, and Cognitive Exercise Training Poststroke. *Stroke, 46*(7), 2012-2016. doi:10.1161/strokeaha.114.006649

van de Port, I. G., Wevers, L. E., Lindeman, E., & Kwakkel, G. (2012). Effects of circuit training as alternative to usual physiotherapy after stroke: randomised controlled trial. *BMJ, 344*, e2672.

Van Der Ploeg, H. P., Streppel, K. R. M., Van Der Beek, A. J., Van Der Woude, L. H. V., Vollenbroek-Hutten, M. M. R., Van Harten, W. H., & Van Mechelen, W. (2006). Counselling increases physical activity behaviour nine weeks after rehabilitation. *British Journal of Sports Medicine, 40*(3), 223-229.

Van Der Ploeg, H. P., Streppel, K. R. M., Van Der Beek, A. J., Van Der Woude, L. H. V., Vollenbroek-Hutten, M. M. R., Van Harten, W. H., & Van Mechelen, W. (2007). Successfully improving physical activity behavior after rehabilitation. *American Journal of Health Promotion, 21*(3), 153-159.

Wan, L. H., Zhang, X. P., Mo, M. M., Xiong, X. N., Ou, C. L., You, L. M., . . . Zhang, M. (2016). Effectiveness of Goal-Setting Telephone Follow-Up on Health Behaviors of Patients with Ischemic Stroke: A Randomized Controlled Trial. *Journal of Stroke and Cerebrovascular Diseases, 25*(9), 2259-2270.

Yelnik, A., Andriantsifanetra, C., Reinert, P., Evrard, M., Marneff, H., Wanepain, M., . . . Vicaut, E. (2016). Active mobility early after stroke. A randomised controled trial (AMOBES). *Annals of Physical and Rehabilitation Medicine, 59*, e67.
